# Impacts of elicitors on metabolite production and on antioxidant potential and tyrosinase inhibition in watercress microshoot cultures

**DOI:** 10.1007/s00253-021-11743-8

**Published:** 2022-01-05

**Authors:** Marta Klimek-Szczykutowicz, Michał Dziurka, Ivica Blažević, Azra Đulović, Anna Apola, Halina Ekiert, Agnieszka Szopa

**Affiliations:** 1 Chair and Department of Pharmaceutical Botany, Faculty of Pharmacy, Jagiellonian University, Medical College, Medyczna 9, 30-688 Kraków, Poland; 2 Department of Dermatology, Cosmetology and Aesthetic Surgery, The Institute of Medical Sciences, Medical College, Jan Kochanowski University, Stefana Żeromskiego 5, 25-369 Kielce, Poland; 3 The Franciszek Górski Institute of Plant Physiology, Polish Academy of Sciences, Niezapominajek 21, 30-239 Kraków, Poland; 4 Department of Organic Chemistry, Faculty of Chemistry and Technology University of Split, Ruđera Boškovića 35, 21000 Split, Croatia; 5 Department of Inorganic Chemistry, Faculty of Pharmacy, Jagiellonian University Medical College, Medyczna 9, 30-688 Kraków, Poland

**Keywords:** *Nasturtium officinale*, In vitro cultures, Elicitation, Glucosinolates, Polyphenols, Antioxidant potential, Tyrosinase inhibition

## Abstract

**Abstract:**

The study has proved the stimulating effects of different strategies of treatments with elicitors on the production of glucosinolates (GSLs), flavonoids, polyphenols, saccharides, and photosynthetic pigments in watercress (*Nasturtium officinale*) microshoot cultures. The study also assessed antioxidant and anti-melanin activities. The following elicitors were tested: ethephon (ETH), methyl jasmonate (MeJA), sodium salicylate (NaSA), and yeast extract (YeE) and were added on day 10 of the growth period. Cultures not treated with the elicitor were used as control. The total GSL content estimations and UHPLC-DAD-MS/MS analyses showed that elicitation influenced the qualitative and quantitative profiles of GSLs. MeJA stimulated the production of gluconasturtiin (68.34 mg/100 g dried weight (DW)) and glucobrassicin (65.95 mg/100 g DW). The elicitation also increased flavonoid accumulation (max. 1131.33 mg/100 g DW, for 100 μM NaSA, collection after 24 h). The elicitors did not boost the total polyphenol content. NaSA at 100 μM increased the production of total chlorophyll *a* and *b* (5.7 times after 24 h of treatment), and 50 μM NaSA caused a 6.5 times higher production of carotenoids after 8 days of treatment. The antioxidant potential (assessed with the CUPRAC FRAP and DPPH assays) increased most after 24 h of treatment with 100 μM MeJA. The assessment of anti-melanin activities showed that the microshoot extracts were able to cause inhibition of tyrosinase (max. 27.84% for 1250 µg/mL).

**Key points:**

*• Elicitation stimulated of the metabolite production in N. officinale microshoots.*

*• High production of pro-health glucosinolates and polyphenols was obtained.*

*• N. officinale microshoots have got tyrosinase inhibition potential.*

*• The antioxidant potential of N. officinale microshoots was evaluated.*

**Graphical abstract:**

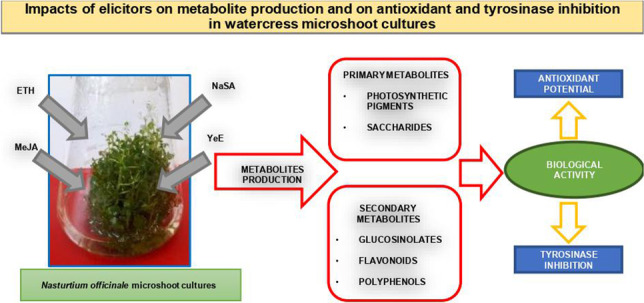

**Supplementary Information:**

The online version contains supplementary material available at 10.1007/s00253-021-11743-8.

## Introduction

*Nasturtium officinale* R. Br., known as watercress, is a perennial, aquatic, or semiaquatic plant species. It grows mainly in wetlands, near streams and running water. *N. officinale* is a widely commercially cultivated species, recently mainly as source of “super food” or “fit food,” due to its low calorific value (11 cal in 100 g of FW (fresh weight)), and a rich source of valuable compounds such as glucosinolates (GSLs), polyphenols, vitamins (B1, B2, B3, B6, E, C), and bioelements (Afsharypuor and Salehi 2008; Martínez-Sánchez et al. 2008; Boligon et al. 2013; Jeon et al. 2017). It is a leafy vegetable, often grown in hydroponic cultures and in gravel beds with a constant flow of water passing through them. It has a short cultivation time. The cultivation takes from 4 to 8 weeks from transplanted seedling to harvest (Lira et al. 2018; De Lira et al. 2019). The European Food Safety Authority (EFSA) has classified *N. officinale* as a safe vegetable under the group “Leaf vegetables, herbs and edible flowers” (EFSA).  Currently, it is becoming one of the most important plants used in healthy food and modern cuisine. *N. officinale* is increasingly being used in European, Brazilian, and North American cuisine. It is commonly used as salad, but also garnish for meats and other dishes where a peppery or pungent flavor is desired (Palaniswamy and McAvoy 2001). Interest in *N. officinale* as a salad vegetable for health promotion and disease prevention has increased due to numerous studies confirming that isothiocyanates in this species reduce the risk of cancer (De Souza et al. 2016; Li et al. 2016; Yuan et al. 2016). Isothiocyanates are compounds formed by the hydrolysis of GSLs *via* myrosinase (Palaniswamy and McAvoy 2001). The conducted studies have also shown that, apart from anticancer activity, extracts of *N. officinale* also have antioxidant, antibacterial, and antiinflammatory properties (Holst and Williamson 2004; Bahramikia and Yazdanparast 2010; Sadeghi et al. 2014; Li et al. 2016).

Plant biotechnology gives possibilities to cultivate rare and protected plants regardless of climatic conditions, with biomass being available all year round. Plant in vitro cultures can be used to stimulate production of valuable secondary metabolites by the influence of various factors on cultures. Finally, they can be cultivated on an industrial scale by being grown in bioreactors (Karuppusamy 2009).

The production of secondary metabolites in plant in vitro cultures can be stimulated by different treatments with elicitors. Elicitation is one of the most promising methods used in plant biotechnology to enhance productivity by stress induction. It involves manipulation of metabolite and biochemical pathways. In plant physiology, “stress” is defined by a factor (biotic or abiotic) which can modify plant growth, reproduction, and functioning. Abiotic elicitors have non-biological sources, which could include both physical agents (e.g., ultraviolet irradiation, temperature, mechanical wounding) and chemical substances, such as salicylic acid (SA), jasmonic acid (JA), and methyl jasmonate (MeJA). Biotic elicitors have a biological origin, i.e., they come from a microbial or plant source, such as yeast extract (YeE). Adding elicitors exogenously to culture media generally causes stimulation of secondary metabolite production under in vitro conditions (Narayani and Srivastava 2017). Studies have shown an increase of the production of oleanolic acid in cell suspension cultures of *Calendula officinalis* after treatments with JA (Wiktorowska et al. 2010). MeJA has been found to induce higher accumulation of resveratrol in cell suspension cultures of *Vitis vinifera* (Santamaria et al. 2012), gymnemic acid in cell suspension cultures of *Gymnema sylvestre* (Chodisetti et al. 2015), ginsenosides in cell suspension cultures of *Panax ginseng* (Thanh et al. 2005), and rosmarinic acid in cell suspension cultures of *Mentha piperita* (Krzyzanowska et al. 2012). There have been scientific studies which also confirmed the stimulating effect of SA on the production of phenolic acids in cell cultures of *Salvia miltiorrhiza* (Dong et al. 2010) and jaceosidin and syringin in cell suspension cultures of *Saussurea medusa* (Yu et al. 2006)*.* Ethephon (ETH) has been demonstrated to stimulate the production of anthocyanins in hairy root cultures of *Daucus carota* ssp. *sativus* var. *atrorubens* (Barba-Espín et al. 2020) and pseudohypericin in *Hypericum perforatum* shoot cultures (Rao et al. 2010). Studies with YeE have confirmed enhanced production of dibenzocyclooctadiene lignans in *Schisandra chinensis* microshoot cultures (Szopa et al. 2018).

The aim of the present research was to evaluate the influence of treatments with different elicitors: ETH, MeJA, sodium salicylate (NaSA), and YeE, on stimulating the production of GSLs, flavonoids, and polyphenols in the *N. officinale* microshoot in vitro culture model. The influence of the applied elicitation strategies were also assessed in respect of the production of primary metabolites. This study were estimated total soluble saccharides and photosynthetic pigments. Moreover, the antioxidant potential and anti-melanin properties of extracts from *N. officinale* microshoots cultured in vitro were evaluated*.*

## Materials and methods

### Experimental in vitro cultures

Initial microshoot cultures of *N. officinale* were established and maintained as reported previously (Klimek-Szczykutowicz et al. 2019). This study involved cultivating *N. officinale* microshoots in 300 mL Erlenmeyer flasks containing 100 mL of Murashige and Skoog (MS) medium (Murashige and Skoog 1962) with 3% (w/v) sucrose and supplemented with 1 mg/L 6-benzyladenine (BA) and 1 mg/L 1-naphthaleneacetic acid (NAA). The medium composition was identified in our previous research as optimal for the cultivation of microshoots (Klimek-Szczykutowicz et al. 2020b). The inoculum used in this study was composed of 1 g of FW of *N. officinale* microshoots. The cultures were grown on a rotary shaker (Altel, Cracow, Poland) at 140 rpm. The microshoots were grown under continuous exposure to a light-emitting diode (LED) white light (2.75 W/m^2^) at a temperature of 25 ± 2°C. All the experiments involved three series of cultures (*n* = 6).

### Elicitation procedure

Sterile stock solutions of elicitors were added to the experimental cultures on day 10 of the growth period. The culture media contained the following concentrations of elicitors: 25 and 50 µM ETH (Sigma-Aldrich, St. Louis, MO, USA), 50 and 100 µM MeJA (Sigma-Aldrich, St. Louis, MO, USA), 50 and 100 µM NaSA (POCH, SA, Gliwice, Poland), and 1 mg/mL and 3 mg/mL YeE (Sigma-Aldrich, St. Louis, MO, USA). The experimental media and biomass samples were collected 24 h, 48 h, and 4, 6, and 8 days after treatments with the elicitors.

A stock solution of ETH was prepared by dissolving 4.16 mg in 55 mL distilled H_2_O for the concentration of 25 µM and 8.34 mg in 55 mL for 50 µM. The solution was filter-sterilized using a 0.22 µm syringe filter (Millex^®^GP; Merck Millipore, Burlington, MA, USA), and 5 mL was added to the culture medium to obtain proper concentration in the medium.

MeJA was prepared by dissolving 12.95 µL in 5 mL of 96% ethanol for the concentration of 50 µM and 25.90 mg in 5 mL of 96% ethanol for 100 µM. Further dilution was made with distilled H_2_O to 55 mL. The solution was filter-sterilized using a 0.22 µm syringe filter (Millex^®^GP; Merck Millipore, Burlington, MA, USA), and 5 mL was added to the culture medium to obtain proper concentration in the medium.

A stock solution of NaSA was prepared by dissolving 9.24 mg in 55 mL distilled H_2_O for the concentration of 50 µM and 18.48 mg in 55 mL for 100 µM. The solution was filter-sterilized using a 0.22 µm syringe filter (Millex^®^GP; Merck Millipore, Burlington, MA, USA), and 5 mL was added to the culture medium to obtain proper concentration in the medium.

YeE was prepared according to the method of Peltonen et al. (1997). YeE amounts of 1.155 and 3.465 g were diluted in 55 mL distilled H_2_O, autoclaved, and added to the culture medium to obtain the concentrations of 1 and 3 mg/mL.

Microshoots which were grown without elicitors were established as control (C), and on day 10 of the growth period, redistilled sterile H_2_O was added to the flask in the amount corresponding to that used to dissolve the elicitors (5 mL). The control samples were collected at the same time points as those of the experimental cultures.

### Calculation of the growth index

Biomass increments were calculated using the growth index (Gi). Microshoots were collected 24 h, 48 h, and 4, 6, and 8 days after the addition of elicitors, lyophilized (freeze dryer, Labconco Corporation, Kansas City, MO, USA), and dried weight (DW) was weighed. The Gi was calculated using the formula: $\mathrm{Gi}=\frac{{(\mathrm{Dw}}_{1}-{\mathrm{Dw}}_{0})}{{\mathrm{Dw}}_{0}}$, where Dw_1_ is the dry weight of microshoots at the end of the experiment, and Dw_0_ is the dry weight of the inoculum (Grzegorczyk and Wysokińska 2008).

### Sample preparation

Plant material harvested from the tested *N. officinale* microshoot cultures was immediately frozen in liquid N_2_ and lyophilized (freeze dryer, Labconco Corporation, Kansas City, MO, USA). The dry biomass was pulverized (MM400, Retch, Haan, Germany). Samples (0.2 g) were weighed out and extracted twice with 4 mL of methanol (STANLAB, Lublin, Poland) under sonication for 20 min (ultrasonic bath; POLSONIC 2, Warsaw, Poland). Then, the samples were centrifuged (7 min, 2000 × g; MPW-223E centrifuge; MPW, Warsaw, Poland) and filtered (0.22 μm syringe filters; Millex^®^GP; Merck Millipore, Burlington, MA, USA). If not otherwise stated, these extracts were used for further analyses.

### Spectrophotometric analysis of the total GSL pool

Analysis of GSLs was performed with the method of Gallaher et al. (2012) as described in our previous studies (Klimek-Szczykutowicz et al. 2019, 2020b). Briefly, lyophilized and pulverized samples were extracted after myrosinase inactivation. The centrifuged extract was evaporated under N_2_ (TurboVap evaporator, Zymark, Midland, MI, USA), and after reconstitution in H_2_O, the samples were subjected to anion-exchange SPE (Supel-Select SAX, 60 mg, 3 mL, Bellefonte, PA, USA) to clean up. The GSLs were eluted with 4 × 0.25 mL of 0.5 M NaCl then dried under N_2_, and the GSLs were hydrolysed with 1 M sodium hydroxide. After 30 min, the samples were neutralized with concentrated hydrochloric acid. The colorimetric reaction of 2 mM potassium ferricyanide solution (0.4 M phosphate buffer pH 7.0) with the standard or sample solution was monitored in 96-well plates. Absorbance was read at 420 nm (Synergy II, BioTek, Winooski, VT, USA) 2 min after ferricyanide addition. The total GSL content was expressed as sinigrin equivalent in mg of sinigrin/100 g DW. All the technical details were given earlier by Klimek-Szczykutowicz et al. (2019, 2020b).

### Total flavonoid assay

The pool of flavonoids was estimated spectrophotometrically according to Ramos et al. (2017). Forty microliters of 10% AlCl_3_ was mixed with 100 μL of methanolic extract and samples were filled to 1000 μL with 5% acetic acid. After 20 min incubation at room temperature, the samples were aliquoted to 96-well plates and the absorbance was recorded (425 nm, Synergy II, BioTek, Winooski, VT, USA). The sum of flavonoids was calculated as mg rutoside equivalent (RE)/100 g DW. The measurements were done in triplicate (including reagent blanks).

### Total polyphenolic assay

Estimation of the total polyphenolic content was done according to the Singleton method (Singleton et al. 1999) with modifications (Bach et al. 2015). The methanolic extracts were prepared as described in "Sample preparation". Water-diluted Folin-Ciocalteu (F–C) phenol reagent (5/2 v/v, 0.45 mL) was mixed with the sample extracts (100 μL). After 10 min, saturated Na_2_CO_3_ (0.45 mL) was added. The incubation of the samples was continued for 2 h in darkness at 25 °C. Then, the samples were centrifuged and aliquoted to 96-well plates. The absorbance was detected at 760 nm (Synergy II, BioTek, Winooski, VT, USA). The pool of phenolic compounds was calculated as mg gallic acid equivalent (GAL)/100 g DW. The analysis was done in triplicate (including reagent blanks).

### Total soluble saccharide determination

To analyze soluble saccharides, the Dubois et al. (1951) phenol–sulfuric method modified by Bach et al. (2015) was used. Samples (5 mg) were extracted in 1.5 mL of ultra-pure water for 15 min, and then centrifuged at 22 000 × g for 5 min at 15 °C. A 20 μL sample of the supernatant was diluted with 180 μL water. Then, 200 μL of a 5% phenol solution and 1 mL concentrated sulfuric acid were added. Samples were left for 20 min incubation and transferred to 96-well plates. The absorbance was measured at 490 nm. The saccharide content was expressed as glucose (GLU) equivalent.

### Analysis of individual glucosinolate content

Isolation of desulfoglucosinolates (dGSLs) was performed as reported previously (Grosser and van Dam 2017; Blažević et al. 2019) from 100 mg samples of dried plant material. The standard used, sinigrin, was obtained from Sigma-Aldrich (Saint Louis, MO, USA); glucohesperin (**1**), glucohirsutin (**3**), gluconasturtiin (**6**), 4-hydroxyglucobrassicin (**7**), glucobrassicin (**8**), and 4-methoxyglucobrassicin (**9**) were obtained from Phytoplan (Heidelberg, Germany). All other chemicals and reagents were of analytical grade.

Analysis was performed by the UHPLC-DAD-MS/MS method (Ultimate 3000RS with TSQ Quantis MS/MS detector, Thermo Fischer Scientific, Waltham, MA, USA) using a Hypersil GOLD column (3.0 µm, 3.0 × 100 mm, Thermo Fischer Scientific, Waltham, MA, USA). A gradient consisting of solvent A (50 μM NaCl in H_2_O) and solvent B (acetonitrile: H_2_O 30:70 v/v) was applied at a flow rate of 0.5 mL/min as follows: 0.14 min 96% A and 4% B; 7.84 min 14% A and 86% B; 8.96 min 14% A and 86% B; 9.52 min 5% A and 95% B; 13.16 min 5% A and 95% B; 13.44 min 96% A and 4% B; 15.68 min 96% A and 4% B. The column temperature was held at 15 °C, and the injection volume was 2 µL. The system was operated in the positive ion electrospray mode and the electrospray interface was H-ESI operating with a capillary voltage of 3.5 kV at 350 °C. The signals were recorded at 227 nm by a diode array detector (DAD). Quantification of dGSLs was performed using an external calibration curve of pure desulfosinigrin (range from 13.56– 542.50 µM). For each individual dGSL response, factors (RPF) were taken in accordance with the literature: RPF 1.0 for **1** and **4**, 1.1 for **3** and** 5** (Brown et al. 2003), 0.95 for **6**, 0.28 for **7,** 0.29 for **8**, 0.25 for **9** (Wathelet et al. 2004); arbitrary 1.0 for **2** (Supplementary Fig. S1).

### Analysis of polyphenol compounds by HPLC–DAD

The analysis was performed using the HPLC–DAD method described previously (Ellnain-Wojtaszek and Zgórka 1999; Sułkowska-Ziaja et al. 2017). For the estimation, methanolic extracts were used (prepared as described in "Sample preparation"). An HPLC–DAD system (Merck-Hitachi, Merck KGaA, Darmstadt, Germany) and a Purospher RP-18e analytical column (4 × 250 nm, 5 mL; Merck) were used. Elution was done with a mobile phase A (methanol:0.5% acetic acid, 1:4 v/v) and a mobile phase B (methanol). The gradient program was set as follows: 0–20 min, 0% B; 20–35 min, 0–20% B; 35–45 min, 20–30% B; 45–55 min, 30–40% B; 55–60 min, 40–50% B; 60–65 min, 50–75% B; and 65–70 min, 75–100% B. The hold time was 15 min. The other parameters were as follows: temperature 25°C, flow rate 1 mL/min, injection volume 20 μL, and detection wavelength 254 nm. Quantitative analysis was carried out for the following compounds identified previously using the UHPLC-DAD-ESI–MS method (Klimek-Szczykutowicz et al. 2020a): *p*-coumaric acid, ferulic acid, and rutoside (Sigma-Aldrich Co. St. Louis, MO, USA).

### Analysis of photosynthetic pigments

Chlorophylls and carotenoids were estimated spectrophotometrically according to Czyczyło-Mysza et al. (2013). Plant material was extracted with 96% ethanol; centrifuged extract (21,000 × g, 5 min at 15 °C) was transferred to a 96-well micro-plate, and the absorbance was read at 470, 648, and 664 nm (Synergy II, BioTek, Winooski, VT, USA). The concentration of chlorophyll *a*, chlorophyll *b*, total chlorophyll (*a* + *b*), and total carotenoids (c) was calculated with Lichtenthaler and Buschman (2001) equations, taking into account the path length of the micro-well.

### Antioxidant activity assays

#### CUPRAC (CUPric Reducing Antioxidant Capacity) assay

The CUPRAC method (Özyürek et al. 2007) with subsequent modifications (Biesaga-Kościelniak et al. 2014) was used to measure the total antioxidant capacity in the analyzed biomass extracts. Equal volumes of methanolic extracts (prepared as described in "Sample preparation"), 10 mmol/L Cu^2+^, 7.5 mmol/L neocuproine, and 1 mol/L ammonia-acetate buffer (pH 7.0) were sequentially dispensed. The samples were mixed and after 15 min (25°C), incubation the absorbance was recorded at 425 nm (96-well plates, Synergy II, BioTek, Winooski, VT, USA). The antioxidant accumulation was calculated as mmol Trolox equivalent (TE)/100 g DW. The measurements were done in triplicate (including reagent blanks).

#### FRAP (Ferric Reducing Antioxidant Power) assay

The antioxidant capacity of the biomass methanolic extracts was also estimated employing the FRAP method (Benzie and Strain 1996). The reagent consisted of a 10 mmol/L TPTZ (2,4,6-tris(2-pyridyl)-s-triazine) soluted in 40 mmol/L HCl with addition of FeCl_3_·6H_2_O (20 mmol/L) and 300 mmol/L of a pH 3.6 acetate buffer (1:1:10 v/v/v). Fifty microliters of extracts was poured with 150 μL of the prepared reagent. After 5 min incubation, the absorbance of the sample was read at 593 nm (96-well plates, Synergy II, BioTek, Winooski, VT, USA). The measurements were done in triplicate (including reagent blanks).

#### DPPH (2,2-diphenyl-1-picrylhydrazyl) radical-scavenging activity assay

The free radical-scavenging activity of the extracts was estimated with the use of the stable radical DPPH (Blios 1958). Fifty microliters of extracts was mixed with 150 μL of DPPH methanolic solution. The samples were incubated for 60 min, and then, its absorbance was read at 517 nm (96-well plates, Synergy II, BioTek, Winooski, VT, USA). The measurements were performed in triplicate (including reagent blanks).

### Inhibition of tyrosinase

The influence of extracts from *N. officinale* microshoot cultures treated with 100 µM MeJA and collected after 24 h on the inhibition of tyrosinase was assessed using the method developed by Chien et al. (2008) and described in previous work (Sułkowska-Ziaja et al. 2021). For this test, we chose the most abundant microshoots based on phytochemical estimations.

The degree of inhibition was determined by spectrophotometry from the UV spectrum with modifications. The percentage of inhibition was estimated by measuring the absorbance of the tested mixtures at 274 nm. At the same time, the spectra of the entire UV range were monitored to identify any undesirable absorption spectrum that could indicate additional reactions in the analyzed mixtures. The evaluation was carried out in a phosphate buffer solution with pH 6.84, in which the analyzed extracts were dissolved. The prepared tyrosinase control solution contained 350 units of enzyme in 1.0 mL. The inhibiting activity was expressed as a percentage of enzyme inhibition, and the assessment of the force of action was carried out relative to kojic acid. The analyzed mixtures were incubated at 37 °C for 25 min. Subsequently, an absorption spectrum was obtained within the wave-length range of 200–400 nm in which a well-developed maximum was observed at 274 nm. The absorption at the maximum was used to evaluate the inhibiting action of the analyzed extracts or kojic acid (control) on tyrosinase activity (Sigma-Aldrich, St. Louis, MO, USA). The impact of the extracts on the enzyme activity was determined using the formula: (%inhibition) = [1 − (*A* − A_0_)/(*B* − B_0_)] × 100, where *A* is the absorbance of the test sample (with tyrosinase, l = 274 nm)., A_0_ is the absorbance of the test sample (without tyrosinase, l = 274 nm), *B*_*0*_ is the absorbance of the control sample (with tyrosinase, l = 274 nm) and B_0_ is the absorbance of the blank sample (without tyrosinase, l = 274 nm).

## Results

### Microshoot appearance and biomass growth

**Fig. 1:**
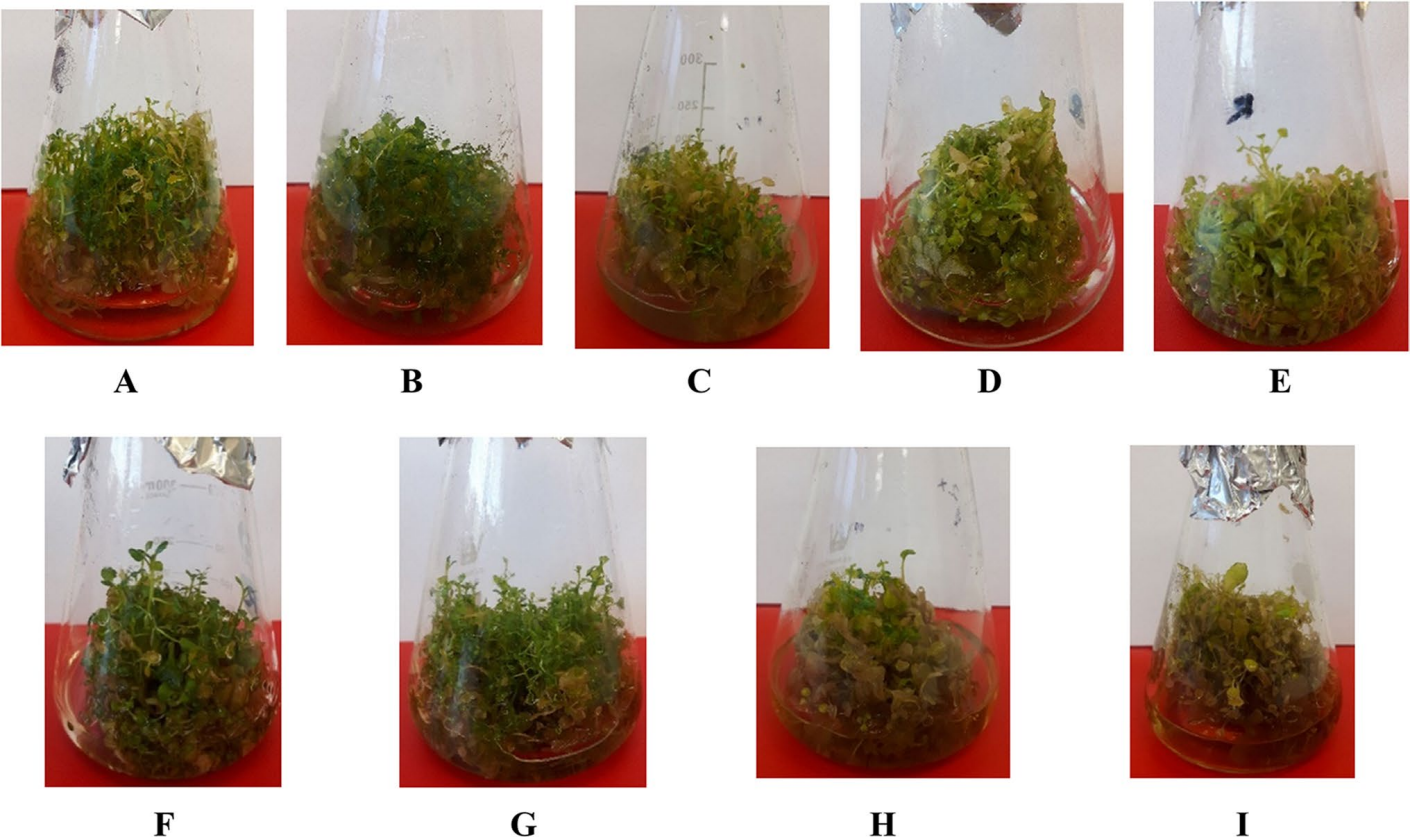
Examples of the morphological appearance of *N. officinale* control and experimental in vitro cultures after 8 days of elicitation. Used concentrations of elicitors: **A** control, **B** 25 µM ETH, **C** 50 µM ETH, **D** 50 µM MeJA, **E** 100 µM MeJA, **F** 50 µM NaSA, **G** 100 µM NaSA, **H** 1 mg/mL YeE, **I** 3 mg/mL YeE. Figure 1 is corresponding with Supplementary Fig. S2

The appearance of *N. officinale* microshoots is affected by the applied elicitors and their concentrations, and also the day of growth after elicitor addition (Supplementary Fig. S2). Microshoots grown with 25 μM ETH were characterized by a green color throughout the entire growth period. The concentration of 50 μM of ETH caused the biomass to die 6 and 8 days after elicitor addition (Fig. 1). In both MeJA concentrations used, the microshoots were smaller and lighter in color 6 and 8 days after elicitor addition (Fig. 1). The microshoots grown with NaSA were vigorous and of a dark green color for the entire period of growth. An increase in the concentration of this elicitor caused no change in the appearance of the cultures. The *N. officinale* cultures grown on MS medium with YeE were characterized by the smallest increase in microshoot biomass at the elicitor concentration of 3 mg/mL. With both YeE concentrations (1 mg/mL and 3 mg/mL), 6 and 8 days after adding the elicitor, the cultures were close to dying (Fig. 1). In control cultures (C), the microshoots were large and green until 4 days after adding the elicitor. After 6 and 8 days, the microshoots became lighter in color (Fig. 1).

The growth index (Gi) for the tested elicitation variants ranged from 6.47 to 31.59. The lowest Gi was obtained with 25 μM ETH for microshoots collected after 24 h of treatment (Table 1 and Supplementary Table S1). The highest Gi was obtained after 6 days of elicitation with 100 μM MeJA. A high Gi was also reached with 50 μM NaSA after 48 h of treatment (Gi = 31.04). The Gi in C ranged from 20.93 to 30.32. The highest Gi for C was obtained after 48 h, and the lowest after 24 h (Table 1 and Supplementary Table S1).

**Table 1:** Maximum values of the growth index (Gi ± SD) for *N. officinale* control and experimental in vitro cultures after elicitation (*p* < 0.05 vs control, *n* = 6)

Elicitor	Gi ± SD maximal value	Elicitor concentration	Harvest time after elicitor treatments	Gi ± SD of C
ETH	30.26 ± 0.03	25 μM	4 days	29.91 ± 0.15
MeJA	31.59 ± 0.01	100 μM	6 days	30.17 ± 0.32
NaSA	31.04 ± 0.07	50 μM	48 h	30.32 ± 0.07
YeE	29.27 ± 0.07	3 mg/mL	48 h	30.32 ± 0.07

^*^Table 1 is corresponding to Supplementary Table S1

### Total and individual GSL content

In order to monitor the total GSL content during plant development, the rapid and cost-effective spectrophotometric method was initially used. The results are given in Supplementary Table S2. The total amount of GSLs was dependent on the addition of the elicitors, their concentrations, and harvesting time. The results of analysis of total GSL content gave us the rationale for choosing the most productive strategies of each elicitor tested: 25 µM ETH (4 days), 50 µM MeJA (24 h), 50 µM NaSA (4 days), and 3 mg/mL YeE (6 days). These samples were subjected to a thorough UHPLC-DAD-MS/MS qualitative and quantitative analyses (Tables 2-3 and Supplementary Fig. S1). In total, nine GSLs were found in the analyzed extracts, namely glucohesperin (**1**), 7-(methylsulfinyl)heptyl GSL (**2**), glucohirsutin (**3**), 7-(methylsulfanyl)heptyl GSL (**4**), 8-(methylsulfanyl)octyl GSL (**5**), gluconasturtiin (**6**), 4-hydroxyglucobrassicin (**7**), glucobrassicin (**8**), and 4-methoxyglucobrassicin (**9**). The amounts of individual compounds in the analyzed extracts ranged from 0.55 to 152.91 mg/100 g DW. The main GSLs according to their amounts were **9**, **8**, and **6.**

**Table 2:** Maximum total glucosinolates (mg/100 g DW ± SD), total flavonoids (mg of RE/100 g DW ± SD), total polyphenols (mg of GAL/100 g DW ± SD), and total soluble saccharides (g of GLU/100 g DW ± SD) for *N. officinale* control and experimental in vitro cultures after elicitation (*p* < 0.05 vs control, *n* = 6)

**Elicitor**	Glucosinolates ^a^			Flavonoids ^b^			Polyphenols ^b^			Soluble saccharides ^b^		
	Maximal total content (mg/100 g DW ± SD)	Elicitor concentration	Harvest time after elicitor treatment	Maximal total content (mg/100 g DW ± SD)	Elicitor concentration	Harvest time after elicitor treatment	Maximal total content (mg/100 g DW ± SD)	Elicitor concentration	Harvest time after elicitor treatment	Maximal total content (g/100 g DW ± SD)	Elicitor concentration	Harvest time after elicitor treatment
C	234.35 ± 14.71	nt*	4 days	984.83 ± 26.72	nt	4 days	336.89 ± 8.03	nt	6 days	7.62 ± 0.46	nt	8 days
ETH	150.71 ± 11.83	25 μM	4 days	1060.80 ± 60.75	50 μM	48 h	314.27 ± 19.20	25 μM	24 h	6.96 ± 0.35	50 μM	24 h
MeJA	211.19 ± 14.04	50 μM	24 h	1037.47 ± 26.80	100 μM	24 h	276.29 ± 19.66	100 μM	24 h	8.30 ± 0.48	100 μM	48 h
NaSA	66.18 ± 7.20	50 μM	4 days	1131.33 ± 9.85	100 μM	24 h	293.21 ± 13.55	50 μM	8 days	9.23 ± 0.34	50 μM	48 h
YeE	11.45 ± 0.91	3 mg/mL	6 days	824.18 ± 25.57	1 mg/mL	8 days	265.89 ± 7.61	1 mg/mL	4 days	6.53 ± 0.28	3 mg/mL	24 h

^*^nt, no treatment; ^a^Calculated from the content of individual GSLs given in Table 3; ^b^Results are corresponding to Supplementary Table S2

**Table 3:** Qualitative and quantitative (mg/100 g DW ± SD) profiles of GSL compounds detected in *N. officinale* control and selected experimental in vitro cultures after elicitation, as confirmed by UHPLC-DAD-MS/MS

Subgroups	No.*	Glucosinolate (GSL) (trivial name)	*t*_R_ (min)	[M + Na]^+^	Contents (mg/100 g DW ± SD)				
					C 4 days	25 μM ETH 4 days	50 μM MeJA 24 h	50 μM NaSA 4 days	3 mg/mL YeE 6 days
Methionine derived	1	6-(Methylsulfinyl)hexyl GSL (glucohesperin)	5.50	408	nd	tr	nd	nd	nd
	2	7-(Methylsulfinyl)heptyl GSL	6.55	422	tr	tr	tr	tr	tr
	3	8-(Methylsulfinyl)octyl GSL (glucohirsutin)	7.58	436	tr	tr	tr	tr	nd
	4	7-(Methylsulfanyl)heptyl GSL	10.97	406	tr	nd	tr	tr	nd
	5	8-(Methylsulfanyl)octyl GSL	12.70	420	tr	nd	nd	tr	nd
Phenylalanine derived	6	2-Phenylethyl GSL (gluconasturtiin)	8.20	366	23.16 ± 4.16	42.79 ± 4.83	68.34 ± 5.14	7.17 ± 0.19	tr
Tryptophan derived	7	4-Hydroxyindol-3-ylmethyl GSL (4-hydroxyglucobrassicin)	5.85	407	12.00 ± 1.89	1.36 ± 0.10	5.26 ± 1.57	0.55 ± 0.00	nd
	8	Indol-3-ylmethyl GSL (glucobrassicin)	7.64	391	46.28 ± 3.20	34.36 ± 0.67	65.95 ± 0.49	4.15 ± 0.12	tr
	9	4-Methoxyindol-3-ylmethyl GSL (4-methoxyglucobrassicin)	8.35	421	152.91 ± 5.46	72.20 ± 6.23	71.64 ± 6.84	54.31 ± 6.89	11.45 ± 0.91

*[M* + *Na]*^+^, sodium adduct of desulfoglucosinolate; *tr*, trace amounts < 0.1 mg/100 g DW; *nd*, not detected

^*^Numbers are corresponding to Supplementary Fig. S1

### Total and individual flavonoid and polyphenol contents

The study has confirmed the influence of the kind of elicitor, its concentration, and the duration of growth on the total amount of flavonoids in extracts of *N. officinale* microshoots. The total amount of flavonoids ranged from 305.86 to 1131.33 mg rutoside equivalent (RE)/100 g DW. The lowest flavonoid content was estimated for C collected after 48 h. The highest flavonoid content was obtained after 24 h of elicitation with 100 μM NaSA (1131.33 mg RE/100 g) and after 8 days of elicitation with 50 μM NaSA (1129.14 mg RE/100 g DW). A high flavonoid content was also estimated for cultures elicited with ETH at 25 μM (1012.47 mg RE/100 g DW) and 50 μM (1060.80 mg RE/100 g DW), and collected after 4 days and 48 h, respectively. The YeE showed the least impact on the production of flavonoids in the analyzed *N. officinale* microshoots. The maximum content for 1 mg/mL YeE (824.18 mg RE/100 g DW) and 3 mg/mL YeE (633.58 mg RE/100 g DW) was obtained after 8 days and 24 h, respectively. The highest amount of flavonoids after elicitation with MeJA at 50 μM (904.36 mg/100 g DW) and 100 μM (1037.47 mg RE/100 g DW) was obtained after 4 days and 24 h, respectively (Table 2 and Supplementary Table S2).

The total amounts of polyphenols in *N. officinale* microshoots ranged from 131.89 to 336.89 mg GAL/100 g DW. The highest polyphenol content was estimated for C microshoots collected after 6 days. The maximal total polyphenol content for the elicited cultures obtained in microshoots treated with ETH was a little lower than for C (for 25 μM, 314.27 mg GAL/100 g DW and for 50 μM, 293.91 mg GAL/100 g DW, for samples collected after 24 h and 6 days, respectively). For elicitation with 50 and 100 μM NaSA, the maximum amounts of polyphenols (293.21 and 268.30 mg GAL/100 g DW) were confirmed after 8 and 4 days of treatment. For elicitation with 1 and 3 mg/mL YeE, the maximum contents were lower than for all the other elicitors. The maximum values of 265.89 and 255.84 mg GAL/100 g DW, respectively, were obtained after 4 days of treatments. The maximum amounts of polyphenols for 50 μM MeJA (269.61 mg GAL/100 g DW) and 100 μM MeJA (276.29 mg GAL/100 g DW) were estimated in microshoots collected after 4 and 8 days, respectively (Table 2 and Supplementary Table S2).

Under our former study (Klimek-Szczykutowicz et al. 2020a), using the UHPLC-DAD-MS/MS method, we confirmed the presence of individual phenolic compounds in *N. officinale* microshoot extracts. Within this study, we estimated quantitatively two phenolic acids (*p*-coumaric and ferulic) and one flavonoid (rutoside) with the HPLC–DAD method (Table 4 and Supplementary Table S3). The amount of *p*-coumaric acid ranged from 1.80 to 64.38 mg/100 g DW. The lowest content was obtained for microshoots collected after 24 h of treatment with 50 μM ETH. The highest content was estimated for samples from C harvested after 8 days. In elicitor-treated samples, the maximum value for *p*-coumaric acid (38.76 mg/100 g DW) was obtained after elicitation with 100 μM MeJA (after 6 days) (Table 4 and Supplementary Table S3). The amount of ferulic acid varied from 1.39 to 17.76 mg/100 g DW. The lowest content was obtained in the C after 48 h. The highest content was found in microshoots collected after 8 days of elicitation with 50 μM NaSA. In C, the maximum value for ferulic acid (16.46 mg/100 g DW) was obtained after 8 days (Table 4 and Supplementary Table S3). The amount of rutoside in extracts from the analyzed *N. officinale* microshoot cultures ranged from 1.14 to 21.17 mg/100 g DW. The lowest content was obtained in microshoot extracts collected after 48 h from cultures treated with 25 μM ETH. The maximum value was obtained for C after 6 days. In extracts from the treated *N. officinale* microshoots, the highest content was obtained after 8 days of treatment with 100 μM NaSA (Table 4 and Supplementary Table S3).

**Table 4:** Maximum amounts of the main phenolic compounds (mg/100 g DW ± SD) in extracts from *N. officinale* control and experimental in vitro cultures after elicitation (*p* < 0.05 vs control, *n* = 6)

Polyphenol compounds	Maximal content (mg/100 g DW ± SD)	Elicitor	Elicitor concentration	Harvest time after elicitor treatment	C (mg/100 g DW ± SD)
*p*-Coumaric acid	38.76 ± 0.72	MeJA	100 μM	6 days	64.38 ± 9.26
Ferulic acid	17.76 ± 2.07	NaSA	50 μM	8 days	16.46 ± 0.09
Rutoside	17.00 ± 1.45	NaSA	100 μM	8 days	21.17 ± 2.67

^*^Table 4 is corresponding to Supplementary Table S3

### Total soluble saccharide content

The total amounts of soluble saccharides ranged from 1.97 to 9.23 g GLU/100 g DW (Table 2 and Supplementary Table S2). For C, the highest amount of soluble saccharides (7.62 g GLU/100 g DW) was obtained after 8 days. The lowest amount of soluble saccharides was obtained after 6 days of treatment with 1 mg/mL YeE. In comparison with C, the total soluble saccharide content increased after elicitation with 50 μM NaSA and 100 μM MeJA after 48 h of treatment (Table 2 and Supplementary Table S2). The highest content was obtained with 50 μM NaSA after 48 h of elicitation (9.23 g GLU/100 g DW) and for 100 μM MeJA after 48 h (8.30 g GLU/100 g DW).

### Photosynthetic pigment contents

The analysis has proven the influence of elicitors on the production of photosynthetic pigments in extracts from *N. officinale* microshoot cultures. The amount of chlorophyll *a* ranged from 4.38 to 89.01 mg/100 g DW. The lowest value was obtained after 48 h for C. The maximum content was in the microshoots collected after 24 h from the cultures supplemented with 100 μM NaSA. The high value (85.56 mg/100 g DW) for chlorophyll *a* was obtained also in C after 4 days (Table 5 and Supplementary Table S4).

**Table 5:** Maximum amounts of photosynthetic pigments (mg/100 g DW ± SD) in extracts from *N. officinale* control and experimental in vitro cultures after elicitation (*p* < 0.05 vs control, *n* = 6)

Photosynthetic pigments	Maximal content (mg/100 g DW ± SD)	Elicitor	Elicitor concentration	Harvest time after elicitor treatment	C (mg/100 g DW ± SD)
Chlorophyll *a*	89.01 ± 2.53	NaSA	100 μM	24 h	12.33 ± 0.33
Chlorophyll *b*	58.25 ± 1.87	NaSA	100 μM	24 h	13.31 ± 0.41
Chlorophyll *a* + *b*	147.32 ± 4.48	NaSA	100 μM	24 h	25.64 ± 0.73
Carotenoids	10.83 ± 1.14	NaSA	50 μM	8 days	1.67 ± 0.06

^*^Table 5 is corresponding to Supplementary Table S4

The amount of chlorophyll *b* in extracts from the experimental *N. officinale* cultures varied from 1.56 to 58.25 mg/100 g DW. The lowest content was obtained after 48 h for C. The maximum value was estimated for microshoots collected 24 h after treatment with 100 μM NaSA (Table 5 and Supplementary Table S4).

The total value of chlorophylls *a* and *b* in the analyzed extracts ranged from 5.99 to 147.32 mg/100 g DW. The lowest content was in C after 48 h. The maximum value was obtained for 100 μM NaSA-elicited microshoots collected after 24 h. A high total value of chlorophylls a and *b* (141.50 mg/100 g DW) was estimated also in C after 4 days (Table 5 and Supplementary Table S4).

The amount of carotenoids in extracts of the experimental *N. officinale* cultures varied from 0.00 to 10.83 mg/100 g DW. The lack of carotenoids was shown for microshoots from C collected after 6 days. The highest content was obtained in the biomass harvested after 8 days of treatment with 50 μM NaSA. In C, the maximum amounts of carotenoids (9.33 mg/100 g DW) were obtained after 4 days (Table 5 and Supplementary Table S4).

### Antioxidant potential

The three assays, CUPRAC, DPPH, and FRAP, were used to estimate the antioxidant potential of *N. officinale* microshoot extracts after elicitation. The antioxidant activity of biomass extracts analyzed with the CUPRAC method ranged from 0.84 to 2.97 mmol TE/100 g DW. The lowest antioxidant activity was shown by C samples collected after 48 h. The maximum antioxidant activity estimated with the CUPRAC method was obtained for microshoots collected after 24 h of elicitation with 100 μM MeJA (Table 6). In the case of C, the highest antioxidant potential (2.61 mmol TE/100 g DW) was obtained after 4 days (Supplementary Table S5).

**Table 6:** Maximum antioxidant activity, estimated by the CUPRAC, DPPH, and FRAP methods (expressed in mmol TE/100 g DW ± SD), of extracts from *N. officinale* control and experimental in vitro cultures after elicitation (*p* < 0.05 vs control, *n* = 6)

Polyphenol compounds	Maximal antioxidant activity	Elicitor	Elicitor concentration	Harvest time after elicitor treatment	C
CUPRAC	2.97 ± 0.03	MeJA	100 μM	24 h	1.63 ± 0.06
DPPH	2.30 ± 2.09	ETH	50 μM	6 days	1.04 ± 0.01
FRAP	0.83 ± 0.02	MeJA	100 μM	24 h	0.40 ± 0.01

^*^Table 6 is corresponding to Supplementary Table S5

The antioxidant activity of experimental biomass extracts estimated with the DPPH method ranged from 0.53 to 2.30 mmol TE/100 g DW. The lowest antioxidant activity was obtained for microshoots treated with 50 μM ETH and collected after 24 h. The maximum antioxidant activity estimated with the DPPH method was obtained after elicitation of microshoots with 50 µM ETH for 6 days (Table 6). In the case of C samples, the highest antioxidant potential (1.19 mmol TE/100 g DW) was shown by microshoots collected after 24 h (Supplementary Table S5).

The antioxidant activity of biomass extracts estimated with the FRAP method ranged from 0.15 to 0.83 mmol TE/100 g DW. The lowest antioxidant activity was shown by extracts from the cultures treated with 3 mg/mL YeE and collected after 8 days. The maximum antioxidant activity was obtained after elicitation with 100 μM MeJA for 24 h (Table 6). In C samples, the highest antioxidant potential (0.75 mmol TE/100 g DW) was shown after 4 days (Supplementary Table S5).

### Tyrosinase inhibition potential

Under this study, we decided to check the possibility of using the analyzed extracts in the treatment of hypermelanosis. The tests of tyrosinase inhibition were performed on extracts of *N. officinale* microshoot cultures with a high antioxidant potential and high flavonoid and polyphenol contents (cultures treated with 100 μM MeJA and collected after 24 h of elicitation). The results showed increased antityrosinase activity with increasing concentration of the tested extract. The inhibition ranged from 5.41 to 27.84%. The highest inhibition was obtained for a concentration of dry extract equal to 1250 µg/mL, and it was similar to tyrosinase inhibition with 5.5 μg/mL of kojic acid. The lowest inhibition was shown by extracts with a concentration of 250 µg dry extract/mL (Table 7).

**Table 7:** Inhibition of tyrosinase activity (% ± SD) by the extracts obtained from elicitor-treated *N. officinale* microshoot cultures (*p* < 0.05 vs control, *n* = 6)

*N. officinale* microshoot culture extract		Positive control	
***N. officinale*** extract (µg dry extract/mL)	Inhibition of tyrosinase activity	Kojic acid (µg)	Inhibition of tyrosinase activity
1250	27.84 ± 0.40	5.5	30.62 ± 0.36
1000	22.03 ± 0.30	4.5	24.93 ± 0.07
750	16.69 ± 0.22	3.5	20.69 ± 0.15
500	11.05 ± 0.16	2.5	13.65 ± 0.06
250	5.41 ± 0.06	1.5	8.54 ± 0.04

## Discussion

The influence of the applied elicitations on the biomass growth and appearance was assessed. In general, the elicitors used did not inhibit biomass growth. In our earlier studies, the highest Gi had been obtained for elicitor non-treated *N. officinale* agitated cultures (Gi = 10.48) collected after 20 days of growth. That value was 3.0 times lower than the highest result obtained after the current treatments with elicitors (max. Gi = 31.04) (Klimek-Szczykutowicz et al. 2020b). The highest values of Gi obtained under our study were comparable to those reached by *N. officinale* microshoots maintained in special temporary immersion systems—RITA^®^ bioreactors—over 20-day growth periods (Gi = 31.71) (Klimek-Szczykutowicz et al. 2020a) (Table 1 and Supplementary Table S1).

The results of analysis of total GSL content gave us the rationale for choosing the most productive strategies of each elicitor tested (Supplementary Table S2). Based on UHPLC-DAD-MS/MS qualitative and quantitative analyses in the C sample, the dominant compound was **9**, while elicitation with 25 μM ETH and 50 μM MeJA caused respectively 1.8 and 3.0 times higher accumulation of **6** in comparison with C. MeJA-treated microshoots contained 1.4 times higher and 2.1 times lower amounts of glucobrassicin and 4-methoxyglucobrassicin, respectively, in comparison with C (Table 3, Supplementary Fig. S1). UHPLC-DAD-MS/MS qualitative and quantitative analyses of *N. officinale* microshoot cultures grown in RITA® bioreactor had been performed by us also formerly (Klimek-Szczykutowicz et al. 2020a). There are noticeable differences between those results and the results for the elicitor-treated *N. officinale* microshoot cultures. The elicitors influenced both the qualitative and quantitative compositions of GSLs. In the bioreactor cultures, the dominant compound was Phe-derived **6** (182.93 mg/100 g DW), which was 2.7-fold higher than in the MeJA elicitor-treated culture, where the maximum content was detected (68.34 mg/100 g DW). Contrary to the bioreactor cultures, **6** was not the main GSL, i.e., the elicitor treated cultures showed the higher production of indole-type GSLs (Trp-derived).

In a study by Rubin et al. (2018), the amounts of two GSLs (**6** and glucotropaeolin) had been investigated in *N. officinale* agar microshoot cultures grown with chitosan, casein hydrolysate, and coconut water as elicitors. In that study, the highest amount of **6** had been obtained in the C (140.40 mg/100 g FW), while after treatment elicitors, the amount of **6** decreases with the maximum amount obtained for 0.5 g/L casein hydrolysate (68.00 mg/100 g FW). The influence of elicitors on the GSL content had also been the object of a study by Sánchez-Pujante et al. (2020) on *Brassica oleracea* L. var. *italica* suspension cell cultures. In their work, they had used coronatine (0.5 and 1 μM) and MeJA (50 and 100 μM). The cultures had been grown with the elicitors for 72 h and MeJA concentration of 50 μM had been chosen as the best elicitor that boosted the production of GSLs by *B. oleracea* cells. In our study, over a similar duration of elicitation treatment (48 h and 4 days), we obtained promising results for 50 μM MeJA, too (Supplementary Table S2). The work by Sánchez-Pujante et al. (2017) had also demonstrated a higher production of **8** in cells grown with 50 μM MeJA in comparison with the control, which was also observed in our experiment (Table 3). In *B. oleracea* cells, MeJA had caused a higher production of **7** and **9** in comparison to the control cells. In our *N. officinale* microshoots, the amounts of these compounds after elicitation were lower than in C (Table 3).

The elicitation treatment influenced the total amount of flavonoids in our *N. officinale* microshoot cultures. The highest total flavonoid content was obtained for microshoot extracts collected after 24 h of treatment with 100 μM NaSA, which was 1.1 times higher than in C. In the case of total polyphenol content determined by the F–C method in the *N. officinale* microshoot cultures treated with elicitors, no stimulation of the production of that group of compounds was observed (Table 2 and Supplementary Table S2).

There have been numerous studies focused on the stimulation of the production of polyphenol compounds in different plant cultures in vitro with the use of different elicitors. For example, in the *Phyllanthus pulcher* callus cultures, SA had caused a higher increase in the production of polyphenols and flavonoids in comparison with MeJA (Danaee et al. 2015). The same effect was observed in our experiments for NaSA (Table 2 and Supplementary Table S2). In extracts from *Salvia virgata* shoot cultures, an increase had been obtained in the total phenolic and flavonoid accumulation after treatments with MeJA and YeE (Attaran Dowom et al. 2017). In the *N. officinale* microshoot cultures, this effect was observed only for the total flavonoids after treatment with MeJA. After elicitation with YeE, the total amount of polyphenols and flavonoids decreased in our study (Table 2 and Supplementary Table S2). Stimulation of the production of total flavonoids and total polyphenols has also been demonstrated for cell suspension cultures of *Phoenix dactylifera* (Al-Khayri and Naik 2020), *Orostachys cartilaginous* (Wen et al. 2019), and shoot cultures of *Artemisia aucheri* (Abbaspour and Ehsanpour, 2016) after treatment with SA as elicitor. In extracts from *B. oleracea* cell suspension cultures, the total amount of polyphenols also increased after elicitation with MeJA (Sánchez-Pujante et al. 2020).

The qualitative and quantitative analyses of polyphenol compounds using the HPLC method also confirmed the low influence of the elicitors on their amounts. In extracts from the elicitor-treated microshoots, the maximum amounts of *p*-coumaric acid and rutoside were respectively 1.7 and 1.2 times lower than in C. Only the maximum amount of ferulic acid was 1.1 times higher after elicitation than in C (Table 4 and Supplementary Table S3). The qualitative and quantitative compositions of polyphenol compounds after treatments with elicitors have also been determined using the HPLC method in *Centella asiatica* shoot cultures (Skrzypczak-Pietraszek et al. 2019). That study had demonstrated different influences of the tested elicitors (MeJA and ETH), on the accumulation of phenolic compounds. The authors had claimed that different elicitors could have different effects on the production of individual phenolic compounds, which was confirmed also in our study (Table 4 and Supplementary Table S3). A study on *Momordica dioica* cell cultures had demonstrated that the elicitors SA and JA might not affect the production of some polyphenolic compounds (Chung et al. 2017). As in our study, higher amounts of *p*-coumaric acid had been found in C. On the other hand, SA and JA stimulated the production of ferulic acid (Table 4 and Supplementary Table S3).

The elicitation treatments applied in this study proved to also influence the amounts of soluble saccharides. The highest saccharide content was obtained for NaSA, which was 1.2 times higher than in C. An increase in the polysaccharide content caused by elicitation with SA has also been reported for *O. cartilaginous* cell cultures (Wen et al. 2019). On the other hand, an increase in soluble saccharides has also been demonstrated for *A. aucheri* shoot cultures treated with SA (Abbaspour and Ehsanpour 2016). Treatments with MeJA performed on *B. oleracea* var. *botrytis* grown in vivo have also been found to increase the soluble saccharide content (Sirhindi et al. 2020).

Under our study on *N. officinale* microshoot cultures, the stimulating effect of elicitation on chlorophyll production was evident after 24 h of treatment with 100 μM NaSA (compared to C, 7.2 and 4.4 times increase in chlorophyll *a* and *b* content, respectively). Moreover, NaSA in both tested concentrations caused, over 6.0 times higher production of carotenoids after 8 days of treatment, in comparison with C (Table 5 and Supplementary Table S4). Similarly, higher amounts, in comparison with the control, for chlorophyll *a* and *b* content had been shown before for *P. dactylifera* and *A. aucheri* shoot cultures treated with SA (Abbaspour and Ehsanpour 2016; Al-Mayahi 2016). Additionally, higher accumulation of carotenoids after elicitation with SA had also been reported for *A. aucheri* cultures (Abbaspour and Ehsanpour 2016). The effect of higher production of carotenoids in in vitro cultures caused by elicitation with SA has also been demonstrated for *M. dioica* cell cultures (Chung et al. 2017). Moreover, the stimulating influence of elicitors (MeJA, chitosan, and YeE) on the total carotenoid content has also been proved in *Cleome rosea* callus cultures (Silva da Rocha et al. 2015).

The influence of elicitation strategies on antioxidant potential assessed with the CUPRAC and FRAP methods showed that the maximum antioxidant activity was obtained after 24 h of treatment with 100 μM MeJA, with the values being 1.8 and 2.1 times higher, respectively, than in C. This phenomenon has also been reported for *A. aucheri* shoot cultures treated with SA (Abbaspour and Ehsanpour 2016). With the DPPH assay, the maximum antioxidant activity was obtained after 6 days of elicitation with 50 μM ETH, and the result was 2.2 times higher than in C (Table 6 and Supplementary Table S5). All the results for antioxidant capacity suggested a considerable influence of the flavonoid and polyphenol contents on this activity.

Under this study, we decided to check the possibility of using the analyzed extracts in the treatment of hypermelanosis. This is the first report confirming anti-melanin activities of *N. officinale* microshoot extracts. Our findings suggest potential usefulness of these extracts in treating dermatological hyperpigmentation problems. Anti-melanin activities had also been studied before for other elicitor-treated in vitro cultures such as *O. cartilaginous* bioreactor cultures elicited with 100 μM SA over a 25-day growth period and harvested after 48 h of treatment which showed the maximum inhibitory concentration for 1400 μg/mL (61.7%) (Wen et al. 2019).

To sum up, our study has proven for the first time the impact of different elicitation strategies with *N. officinale* agitated microshoot cultures for the production of GSLs, flavonoids, polyphenols, saccharides, and photosynthetic pigments. Moreover, the antioxidant and anti-melanin activities of biomass extracts from these cultures were documented.

The experiments showed differences in the accumulation of GSLs and the stimulating effect of elicitation on the production of individual compounds—gluconasturtiin and glucobrassicin—which are precursors of isothiocyanates well known for their anticancer, antioxidant, and antibacterial properties. Moreover, the elicitors used increased the production of total flavonoids (100 µM NaSA), soluble saccharides (50 µM NaSA), and photosynthetic pigments (100 µM NaSA). The elicitation treatments did not adversely impact biomass growth; even 100 µM MeJA and 50 µM NaSA stimulated microshoot multiplication.

Furthermore, the experiments demonstrated increased antioxidant activity of *N. officinale* microshoot extracts after the applied elicitation treatments. Additionally, for the first time, biomass extracts from *N. officinale* microshoot cultures have been proved to inhibit tyrosinase activity.

## Supplementary Information

Below is the link to the electronic supplementary material.

Supplementary file1 (PDF 456 KB)

## Data Availability

Raw data were generated at the Chair and Department of Pharmaceutical Botany, Faculty of Pharmacy, Jagiellonian University, Medical College. Derived data supporting the findings of this study are available from the corresponding author on request.

## References

[CR1] Abbaspour J, Ehsanpour A (2016) The impact of salicylic acid on some physiological responses of *Artemisia aucheri* Boiss. under in vitro drought stress. Acta Agric Slov 107:287–298 . 10.14720/aas.2016.107.2.03 10.1186/s40529-016-0154-6 PMC5430568 28597449

[CR2] Afsharypuor S, Salehi M (2008). Volatile constituents of leaves and stems of *Nasturtium officinale* R. Br J Essent Oil Res.

[CR3] Al-Khayri JM, Naik PM (2020). Elicitor-induced production of biomass and pharmaceutical phenolic compounds in cell suspension culture of date palm (Phoenix dactylifera L.). Molecules.

[CR4] Al-Mayahi AMW (2016) Influence of salicylic acid (SA) and ascorbic acid (ASA) on in vitro propagation and salt tolerance of date palm (*Phoenix dactylifera* L.) cv. “Nersy.” Aust J Crop Sci 10:969–976 . 10.21475/ajcs.2016.10.07.p7640

[CR5] Attaran Dowom S, Abrishamchi P, Radjabian T, Salami SA (2017). Enhanced phenolic acids production in regenerated shoot cultures of *Salvia virgata* Jacq. after elicitation with Ag^+^ ions, methyl jasmonate and yeast extract. Ind Crops Prod.

[CR6] Bach A, Kapczyńska A, Dziurka K, Dziurka M (2015). Phenolic compounds and carbohydrates in relation to bulb formation in *Lachenalia* “Ronina” and “Rupert” in vitro cultures under different lighting environments. Sci Hortic (Amsterdam).

[CR7] Bahramikia S, Yazdanparast R (2010). Antioxidant efficacy of *Nasturtium officinale* extracts using various in vitro assay systems. JAMS J Acupunct Meridian Stud.

[CR8] Barba-Espín G, Chen ST, Agnolet S, Hegelund JN, Stanstrup J, Christensen JH, Müller R, Lütken H (2020). Ethephon-induced changes in antioxidants and phenolic compounds in anthocyanin-producing black carrot hairy root cultures. J Exp Bot.

[CR9] Benzie IFF, Strain JJ (1996). The ferric reducing ability of plasma (FRAP) as a measuer of “antioxidant power”: the FRAP assay. Anal Biochem.

[CR10] Biesaga-Kościelniak J, Dziurka M, Ostrowska A, Mirek M, Kościelniak J, Janeczko A (2014). Brassinosteroid improves content of antioxidants in seeds of selected leguminous plants. Aust J Crop Sci.

[CR11] Blažević I, Đulović A, Čikeš Čulić V, Burčul F, Ljubenkov I, Ruščić M, Generalić Mekinić I (2019). *Bunias erucago* L.: glucosinolate profile and in vitro biological potential. Molecules.

[CR12] Blios MS (1958). Antioxidant determinations by the use of a stable free radical. Nature.

[CR13] Boligon AA, Janovik V, Boligon AA, Pivetta CR, Pereira RP, Da RJBT, Athayde ML (2013). HPLC analysis of polyphenolic compounds and antioxidant activity in *Nasturtium officinale*. Int J Food Prop.

[CR14] Brown PD, Tokuhisa JG, Reichelt M, Gershenzon J (2003). Variation of glucosinolate accumulation among different organs and developmental stages of *Arabidopsis thaliana*. Phytochemistry.

[CR15] Chien CC, Tsai ML, Chen CC, Chang SJ, Tseng CH (2008). Effects on tyrosinase activity by the extracts of *Ganoderma lucidum* and related mushrooms. Mycopathologia.

[CR16] Chodisetti B, Rao K, Gandi S, Giri A (2015). Gymnemic acid enhancement in the suspension cultures of *Gymnema sylvestre* by using the signaling molecules—methyl jasmonate and salicylic acid. Vitr Cell Dev Biol - Plant.

[CR17] Chung IM, Rekha K, Rajakumar G, Thiruvengadam M (2017). Jasmonic and salicylic acids enhanced phytochemical production and biological activities in cell suspension cultures of spine gourd (*Momordica dioica* Roxb). Acta Biol Hung.

[CR18] Czyczyło-Mysza I, Tyrka M, Marcińska I, Skrzypek E, Karbarz M, Dziurka M, Hura T, Dziurka K, Quarrie SA (2013). Quantitative trait loci for leaf chlorophyll fluorescence parameters, chlorophyll and carotenoid contents in relation to biomass and yield in bread wheat and their chromosome deletion bin assignments. Mol Breed.

[CR19] Danaee M, Farzinebrahimi R, Kadir MA, Sinniah UR, Mohamad R, Mat Taha R (2015). Effects of MeJA and SA elicitation on secondary metabolic activity, antioxidant content and callogenesis in *Phyllanthus pulcher*. Rev Bras Bot.

[CR20] De Lira RM, De Franca E Silva EF, Da Silva AO , De Medeiros PRF, Da Silva GF, Soares HRE (2019) Watercress and chinese cabbage in a hydroponic system using groundwater. Rev Caatinga 32:1038–1047. 10.1590/1983-21252019v32n420rc

[CR21] De Souza DA, Costa PM, Ribeiro RIMA, Vidigal PVT, Pinto FCH (2016). Daily intake of watercress causes inhibition of experimental Ehrlich tumor growth. J Bras Patol e Med Lab.

[CR22] Dong J, Wan G, Liang Z (2010). Accumulation of salicylic acid-induced phenolic compounds and raised activities of secondary metabolic and antioxidative enzymes in *Salvia miltiorrhiza* cell culture. J Biotechnol.

[CR23] Dubois M, Gilles K, Hamilton JK, Rebers PA, Smith F (1951). A colorimetric method for the determination of sugars. Nature.

[CR24] EFSA European Food Safety Authority (EFSA). http://www.efsa.europa.eu/. Accessed 25 Jan 2021

[CR25] Ellnain-Wojtaszek M, Zgórka G (1999). High-performance liquid chromatography and thin-layer chromatography of phenolic acids from *Gingko biloba* L. leaves collected within vegetative period. J Liq Chromatogr Relat Technol.

[CR26] Gallaher CM, Gallaher DD, Peterson S (2012). Development and validation of a spectrophotometric method for quantification of total glucosinolates in cruciferou*s* vegetables. J Agric Food Chem.

[CR27] Grosser K, van Dam NM (2017). A straightforward method for glucosinolate extraction and analysis with high-pressure liquid chromatography (HPLC). J vis Exp.

[CR28] Grzegorczyk I, Wysokińska H (2008). Liquid shoot culture of *Salvia officinalis* L. for micropropagation and production of antioxidant compounds; effects of triacontanol. Acta Soc Bot Pol.

[CR29] Holst B, Williamson G (2004). A critical review of the bioavailability of glucosinolates and related compounds. Nat Prod Rep.

[CR30] Jeon J, Bong SJ, Park JS, Park YK, Arasu MV, Al-Dhabi NA, Park SU (2017). De novo transcriptome analysis and glucosinolate profiling in watercress (*Nasturtium officinale* R. Br.). BMC Genomics.

[CR31] Karuppusamy S (2009). A review on trends in production of secondary metabolites from higher plants by in vitro tissue, organ and cell cultures. J Med Plants Res.

[CR32] Klimek-Szczykutowicz M, Dziurka M, Blažević I, Đulović A, Granica S, Korona-Glowniak I, Ekiert H, Szopa A (2020). Phytochemical and biological activity studies on *Nasturtium officinale* (watercress) microshoot cultures grown in RITA^®^ temporary immersion systems. Molecules.

[CR33] Klimek-Szczykutowicz M, Szopa A, Blicharska E, Dziurka M, Komsta Ł, Ekiert H (2019). Bioaccumulation of selected macro- and microelements and their impact on antioxidant properties and accumulation of glucosinolates and phenolic acids in in vitro cultures of *Nasturtium officinale* (watercress) microshoots. Food Chem.

[CR34] Klimek-Szczykutowicz M, Szopa A, Dziurka M, Komsta Ł, Tomczyk M, Ekiert H (2020). The influence of Nasturtium officinale R Br agar and agitated microshoot culture media on glucosinolate and phenolic acid production, and antioxidant activity. Biomolecules.

[CR35] Krzyzanowska J, Czubacka A, Pecio L, Przybys M, Doroszewska T, Stochmal A, Oleszek W (2012). The effects of jasmonic acid and methyl jasmonate on rosmarinic acid production in *Mentha × piperita* cell suspension cultures. Plant Cell Tissue Organ Cult.

[CR36] Li Q, Zhan M, Chen W, Zhao B, Yang K, Yang J, Yi J, Huang Q, Mohan M, Hou Z, Wang J (2016) Phenylethyl isothiocyanate reverses cisplatin resistance in biliary tract cancer cells via glutathionylation-dependent degradation of Mcl-1. Oncotarget 7:10271–10282 . 10.18632/oncotarget.7171 10.18632/oncotarget.7171 PMC4891119 26848531

[CR37] Lira RM, Silva ÊFF, Silva GF, Soares HR, Willadino LG (2018). Growth, water consumption and mineral composition of watercress under hydroponic system with brackish water. Hortic Bras.

[CR38] Martínez-Sánchez A, Gil-Izquierdo A, Gil MI, Ferreres F (2008). A comparative study of flavonoid compounds, vitamin C, and antioxidant properties of baby leaf *Brassicaceae* species. J Agric Food Chem.

[CR39] Murashige T, Skoog F (1962). A revised medium for rapid growth and bioassays with tobacco tissue cultures. Physiol Plant.

[CR40] Narayani M, Srivastava S (2017). Elicitation: a stimulation of stress in in vitro plant cell/tissue cultures for enhancement of secondary metabolite production. Phytochem Rev.

[CR41] Özyürek M, Güçlü K, Bektaşoğlu B, Apak R (2007). Spectrophotometric determination of ascorbic acid by the modified CUPRAC method with extractive separation of flavonoids–La(III) complexes. Anal Chim Acta.

[CR42] Palaniswamy UR, McAvoy RJ (2001) Watercress: a salad crop with chemopreventive potential. Horttechnology 11:622–626 . 10.21273/horttech.11.4.622

[CR43] Peltonen S, Mannonen L, Karjalainen R (1997). Elicitor-induced changes of phenylalanine ammonia-lyase activity in barley cell suspension cultures. Plant Cell Tissue Organ Cult.

[CR44] Ramos RTM, Bezerra ICF, Ferreira MRA, Soares LAL (2017). Spectrophotometric quantification of flavonoids in herbal material, crude extract, and fractions from leaves of *Eugenia uniflora* Linn. Pharmacognosy Res.

[CR45] Rao UMV, Gaviraj EN, Veeresham C (2010). Effect of ethephon and ancymidol on the production of hypericins in shoot cultures of *Hypericum perforatum*. Indian Drugs.

[CR46] Rubin E, Aziz ZA, Surugau N (2018). Glucosinolates content in non-elicited plant culture, elicited plant culture and wild plant of watercress (*Nasturtium officinale*). Trans Sci Tech.

[CR47] Sadeghi H, Mostafazadeh M, Sadeghi H, Naderian M, Barmak MJ, Talebianpoor MS, Mehraban F (2014). In vivo anti-inflammatory properties of aerial parts of *Nasturtium officinale*. Pharm Biol.

[CR48] Sánchez-Pujante PJ, Borja-Martínez M, Pedreño MÁ, Almagro L (2017). Biosynthesis and bioactivity of glucosinolates and their production in plant in vitro cultures. Planta.

[CR49] Sánchez-Pujante PJ, Gionfriddo M, Sabater-Jara AB, Almagro L, Pedreño MA, Diaz-Vivancos P (2020). Enhanced bioactive compound production in broccoli cells due to coronatine and methyl jasmonate is linked to antioxidative metabolism. J Plant Physiol.

[CR50] Santamaria AR, Innocenti M, Mulinacci N, Melani F, Valletta A, Sciandra I, Pasqua G (2012). Enhancement of viniferin production in *Vitis vinifera* L. cv. Alphonse Lavallée cell suspensions by low-energy ultrasound alone and in combination with methyl jasmonate. J Agric Food Chem.

[CR51] Silva da Rocha A, Rocha EK, Alves LM, Amaral de Moraes B, Carvalho de Castro T, Albarello N, Simões-Gurgel C (2015). Production and optimization through elicitation of carotenoid pigments in the in vitro cultures of *Cleome rosea* Vahl (Cleomaceae). J Plant Biochem Biotechnol.

[CR52] Singleton VL, Orthofer R, Lamuela-Raventos RM (1999). Analysis of total phenols and other oxidation substrates and antioxidants by means of Folin-Ciocalteu reagent. Methods Enzym.

[CR53] Sirhindi G, Kaushik S, Mushtaq R, Sharma P, Dogra N (2020). Jasmonates induce growth and modulation in pigments and vitamins of *Brassica oleracea* var. *capitata*, *italica* and *botrytis* edible heads (foliage/inflorescence). Rev Bras Bot.

[CR54] Skrzypczak-Pietraszek E, Urbańska A, Żmudzki P, Pietraszek J (2019). Elicitation with methyl jasmonate combined with cultivation in the Plantform^TM^ temporary immersion bioreactor highly increases the accumulation of selected centellosides and phenolics in *Centella asiatica* (L.) Urban shoot culture. Eng Life Sci.

[CR55] Sułkowska-Ziaja K, Grabowska K, Apola A, Kryczyk-Poprawa A, Muszyńska B (2021). Mycelial culture extracts of selected wood-decay mushrooms as a source of skin-protecting factors. Biotechnol Lett.

[CR56] Sułkowska-Ziaja K, Maślanka A, Szewczyk A, Muszyńska B (2017). Physiologically active compounds in four species of genus *Phellinus*. Nat Prod Commun.

[CR57] Szopa A, Kokotkiewicz A, Król A, Luczkiewicz M, Ekiert H (2018). Improved production of dibenzocyclooctadiene lignans in the elicited microshoot cultures of *Schisandra chinensis* (Chinese magnolia vine). Appl Microbiol Biotechnol.

[CR58] Thanh NT, Murthy HN, Yu KW, Hahn EJ, Paek KY (2005). Methyl jasmonate elicitation enhanced synthesis of ginsenoside by cell suspension cultures of *Panax ginseng* in 5-l balloon type bubble bioreactors. Appl Microbiol Biotechnol.

[CR59] Wathelet JP, Iori R, Leoni O, Quinsac O, Palmieri S (2004). Guidelines for glucosinolate analysis in green tissues used for biofumigation. Agroindustria.

[CR60] Wen T, Hao YJ, An XL, Sun HD, Li YR, Chen X, Piao XC, Lian ML (2019) Improvement of bioactive compound accumulation in cell cultures of *Orostachys cartilaginous* A. Bor. through elicitation with salicylic acid and effect of cell extract on bioactive activity. Ind Crops Prod 139:111570 . 10.1016/j.indcrop.2019.111570

[CR61] Wiktorowska E, Długosz M, Janiszowska W (2010). Significant enhancement of oleanolic and accumulation by biotic elicitors in cell suspension cultures of *Calendula officinalis* L. Enzyme Microb Technol.

[CR62] Yu ZZ, Fu CX, Han YS, Li YX, Zhao DX (2006). Salicylic acid enhances jaceosidin and syringin production in cell cultures of *Saussurea medusa*. Biotechnol Lett.

[CR63] Yuan J-M, Stepanov I, Murphy SE, Wang R, Allen S, Jensen J, Strayer L, Adams-Haduch J, Upadhyaya P, Le C, Kurzer MS, Nelson HH, Yu MC, Hatsukami D, Hecht SS (2016). Clinical trial of 2-phenethyl isothiocyanate as an inhibitor of metabolic activation of a tobacco-specific lung carcinogen in cigarette smokers. Cancer Prev Res.

