# Supplementary material for: Impacts of elicitors on metabolite production and on antioxidant potential and tyrosinase inhibition in watercress microshoot cultures
Source: Appl Microbiol Biotechnol. 2022 Jan 5;106(2):619–33. doi: 10.1007/s00253-021-11743-8 (PMC8763773; doi:10.1007/s00253-021-11743-8)
Supplement: Supplementary file 1 — Supplementary file1 (PDF 456 KB) [file 253_2021_11743_MOESM1_ESM.docx]

**Applied Microbiology and Biotechnology**

original article

**Impacts of elicitors on metabolite production and on antioxidant potential and tyrosinase inhibition in watercress microshoot cultures**

Marta Klimek-Szczykutowicz^1,2^, Michał Dziurka^3^, Ivica Blažević^4^, Azra Đulović^4^, Anna Apola^5^, Halina Ekiert^1^, Agnieszka Szopa^1*^

^1^Chair and Department of Pharmaceutical Botany, Faculty of Pharmacy, Jagiellonian University, Medical College, Medyczna 9, 30-688 Kraków, Poland; marta.klimek-szczykutowicz@doctoral.uj.edu.pl (M.K.-S.); halina.ekiert@uj.edu.pl (H.E.)

^2^Department of Dermatology, Cosmetology and Aesthetic Surgery, The Institute of Medical Sciences, Medical College, Jan Kochanowski University, Stefana Żeromskiego 5, 25-369 Kielce, Poland

^3^ The Franciszek Górski Institute of Plant Physiology, Polish Academy of Sciences, Niezapominajek 21,30-239 Kraków, Poland; m.dziurka@ifr-pan.edu.pl (M.D.)

^4^Department of Organic Chemistry, Faculty of Chemistry and Technology University of Split, Ruđera Boškovića 35, 21000 Split, Croatia; blazevic@ktf-split.hr (I.B.); azra@ktf-split.hr (A.Đ.)

^5^Department of Inorganic Chemistry, Faculty of Pharmacy, Jagiellonian University Medical College, Medyczna 9, 30-688 Kraków, Poland; anna.apola@uj.edu.pl (A.A.)

*Corresponding author: phone +48 12 620 54 30, fax +48 620 54 40, e-mail: a.szopa@uj.edu.pl (A.S.)


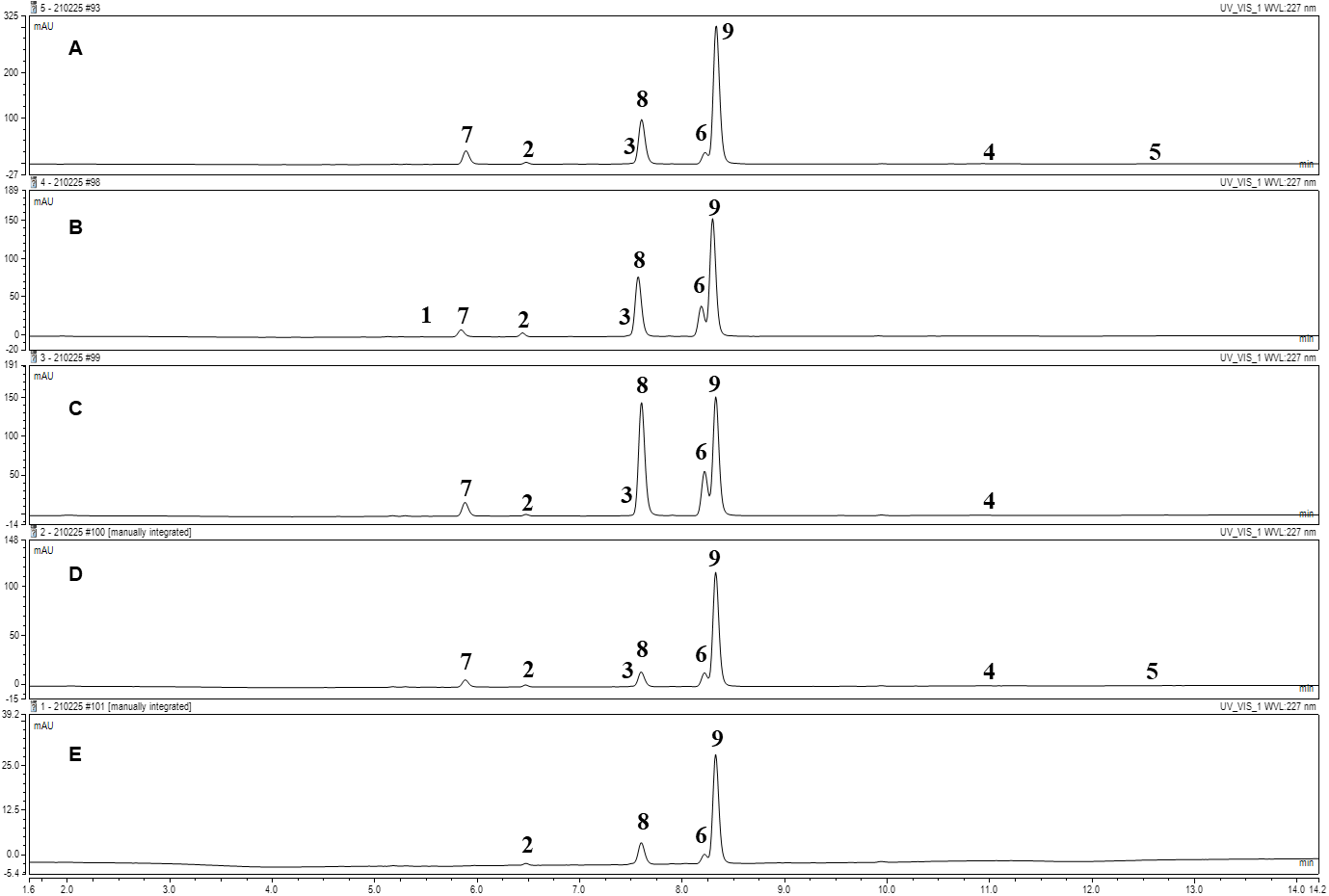


**Figure S1.** Chromatogram of desulfoglucosinolates (dGSLs): **A** – Control (C); **B** – Ethephon (ETH); **C** – Methyl jasmonate (MeJa); **D** – Sodium salicylate (NaSA); **E** – Yeast extract (YeE). Chromatogram of desulfoglucosinolates (dGSL): **1** – 6-(methylsulfinyl)hexyl dGSL; **2** – 7-(methylsulfinyl)heptyl dGSL; **3** – 8-(methylsulfinyl)octyl dGSL; **4** – 7-(methylsulfanyl)heptyl dGSL; **5** – 8-(methylsulfanyl)octyl dGSL; **6** – desulfogluconasturtiin; **7** – 4-hydroxyindol-3-ylmethyl dGSL; **8** – indol-3-ylmethyl dGSL; **9** – 4-methoxyindol-3-ylmethyl dGSL.

| **Elicitor** | **Elicitor concentration** | **Harvest time after elicitor treatments** | | | | |
| --- | --- | --- | --- | --- | --- | --- |
|  |  | **24 h** | **48 h** | **4 d** | **6 d** | **8 d** |
| C | 0 | 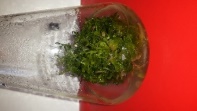 | 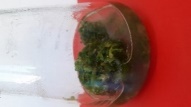 | 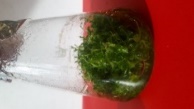 | 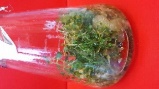 | 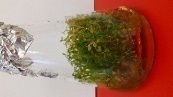 |
| ETH | 25 μM | 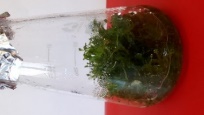 | 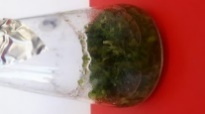 | 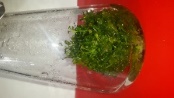 | 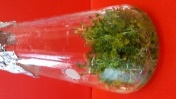 | 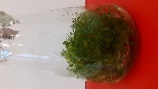 |
|  | 50 μM | 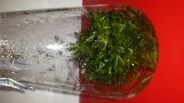 | 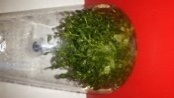 | 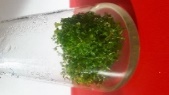 | 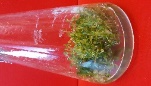 | 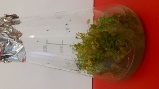 |
| MeJA | 50 μM | 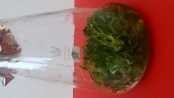 | 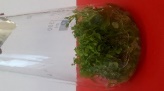 | 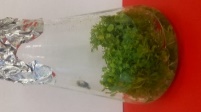 | 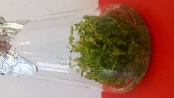 | 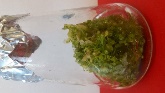 |
|  | 100 μM | 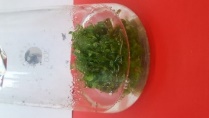 | 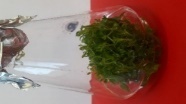 | 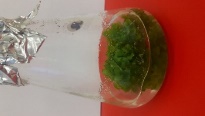 | 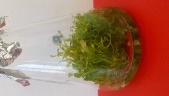 | 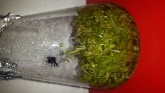 |
| NaSA | 50 μM | 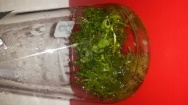 | 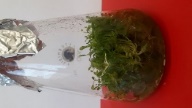 | 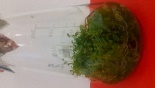 | 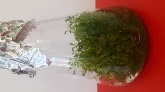 | 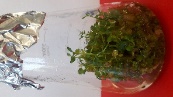 |
|  | 100 μM | 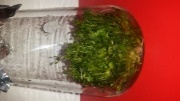 | 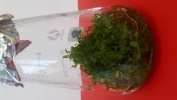 | 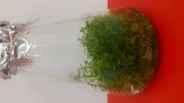 | 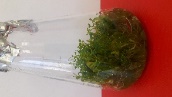 | 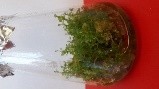 |
| YeE | 1 mg/mL | 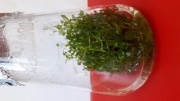 | 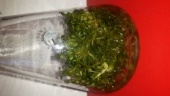 | 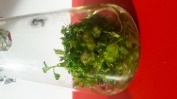 | 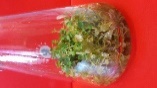 | 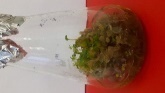 |
|  | 3 mg/mL | 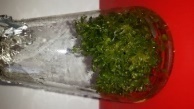 | 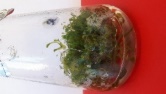 | 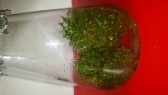 | 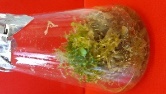 | 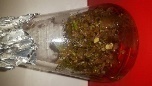 |

**Figure S2.** Morphological appearance of *N. officinale* control and experimental in vitro cultures after elicitation.

**Table S1.** The values of the growth index (Gi ± SD) of *N. officinale* control and experimental in vitro cultures after elicitation (*p* < 0.05 vs control, n=6).

| **Elicitor** | **Elicitor concentration** | **Harvest time after elicitor treatments** | **Gi** |
| --- | --- | --- | --- |
| C | 0 | 24 h | 20.93±0.06 |
|  |  | 48 h | 30.32±0.07 |
|  |  | 4 days | 29.91±0.15 |
|  |  | 6 days | 30.17±0.32 |
|  |  | 8 days | 29.56±0.21 |
| ETH | 25 μM | 24 h | 6.47±0.20 |
|  |  | 48 h | 28.32±0.29 |
|  |  | 4 days | 30.26±0.03 |
|  |  | 6 days | 26.48±0.20 |
|  |  | 8 days | 24.72±0.04 |
|  | 50 μM | 24 h | 12.13±0.43 |
|  |  | 48 h | 28.68±0.29 |
|  |  | 4 days | 27.23±0.04 |
|  |  | 6 days | 26.48±0.21 |
|  |  | 8 days | 24.23±0.05 |
| MeJA | 50 μM | 24 h | 20.21±0.34 |
|  |  | 48 h | 26.11±0.11 |
|  |  | 4 days | 30.58±0.10 |
|  |  | 6 days | 28.03±0.10 |
|  |  | 8 days | 27.77±0.10 |
|  | 100 μM | 24 h | 19.72±0.17 |
|  |  | 48 h | 25.71±0.17 |
|  |  | 4 days | 26.63±0.32 |
|  |  | 6 days | 31.59±0.01 |
|  |  | 8 days | 27.26±0.27 |
| NaSA | 50 μM | 24 h | 30.01±0.02 |
|  |  | 48 h | 31.04±0.07 |
|  |  | 4 days | 29.04±0.09 |
|  |  | 6 days | 30.12±0.08 |
|  |  | 8 days | 27.01±0.13 |
|  | 100 μM | 24 h | 20.55±0.07 |
|  |  | 48 h | 25.93±0.29 |
|  |  | 4 days | 28.42±0.29 |
|  |  | 6 days | 26.81±0.11 |
|  |  | 8 days | 25.35±0.02 |
| YeE | 1 mg/mL | 24 h | 8.50±0.01 |
|  |  | 48 h | 27.57±0.21 |
|  |  | 4 days | 26.51±0.05 |
|  |  | 6 days | 25.23±0.02 |
|  |  | 8 days | 12.34±0.52 |
|  | 3 mg/mL | 24 h | 16.98±0.73 |
|  |  | 48 h | 29.27±0.07 |
|  |  | 4 days | 28.27±0.05 |
|  |  | 6 days | 24.25±0.08 |
|  |  | 8 days | 12.15±0.62 |

**Table S2.** Total glucosinolates (mg of sinigrin/100 g DW), total flavonoids (mg of RE/100 g DW ±SD), polyphenols (mg of GAL/100 g DW) and soluble carbohydrates contents (g of GLU/100 g DW ±SD) of *N. officinale* control and experimental in vitro cultures after elicitation (*p* < 0.05 vs control, n=6).

| **Elicitor** | **Elicitor concentration** | **Harvest time after elicitor treatments** | **Total content of glucosinolates** | **Total content of flavonoids** | **Total content of polyphenols** | **Total soluble saccharides** |
| --- | --- | --- | --- | --- | --- | --- |
|  |  |  | **mg/100 g DW±SD** | **mg/100 g DW±SD** | **mg/100 g DW±SD** | **g/100 g DW±SD** |
| C | 0 | 24 h | 160.41±9.89 | 795.82±3.10 | 261.16±2.42 | 6.97±0.07 |
|  |  | 48 h | 159.12±6.24 | 305.86±31.76 | 167.22±2.56 | 6.76±1.62 |
|  |  | 4 days | 260.66±14.57 | 984.83±26.72 | 296.45±5.26 | 6.39±0.36 |
|  |  | 6 days | 235.05±11.77 | 828.43±36.09 | 336.89±8.03 | 3.54±0.31 |
|  |  | 8 days | 117.75±13.23 | 479.45±3.68 | 252.54±5.43 | 7.62±0.46 |
| ETH | 25 μM | 24 h | 109.99±14.97 | 602.30±26.30 | 314.27±19.20 | 5.34±0.58 |
|  |  | 48 h | 167.50±7.43 | 645.96±27.01 | 187.95±5.16 | 4.37±0.17 |
|  |  | 4 days | 226.31±13.70 | 1012.47±125.45 | 264.87±21.80 | 5.92±0.68 |
|  |  | 6 days | 220.60±6.73 | 549.27±29.88 | 275.32±38.65 | 3.51±0.32 |
|  |  | 8 days | 142.96±8.14 | 733.53±25.35 | 197.41±22.02 | 2.97±0.14 |
|  | 50 μM | 24 h | 137.54±7.96 | 660.15±33.92 | 233.88±19.29 | 6.96±0.35 |
|  |  | 48 h | 176.90±1.22 | 1060.80±60.75 | 239.60±1.19 | 5.92±0.17 |
|  |  | 4 days | 210.43±16.00 | 988.49±50.55 | 290.66±12.52 | 5.33±0.31 |
|  |  | 6 days | 167.44±8.88 | 677.99±20.60 | 293.91±33.70 | 3.45±0.03 |
|  |  | 8 days | 201.57±16.91 | 741.83±30.51 | 193.95±5.63 | 3.14±0.20 |
| MeJA | 50 μM | 24 h | 298.36±15.58 | 858.95±58.66 | 262.35±16.55 | 8.18±0.16 |
|  |  | 48 h | 277.95±14.22 | 779.47±44.40 | 239.92±3.71 | 7.56±0.37 |
|  |  | 4 days | 251.91±18.24 | 904.36±20.82 | 269.61±14.20 | 6.57±0.07 |
|  |  | 6 days | 254.94±20.52 | 606.12±42.00 | 224.75±0.12 | 4.72±0.11 |
|  |  | 8 days | 184.89±15.85 | 580.00±179.98 | 233.08±44.58 | 5.42±1.28 |
|  | 100 μM | 24 h | 297.21±21.13 | 1037.47±26.80 | 276.29±19.66 | 7.52±0.33 |
|  |  | 48 h | 259.85±8.65 | 967.13±24.57 | 264.75±16.48 | 8.30±0.48 |
|  |  | 4 days | 151.87±15.46 | 682.09±30.87 | 224.19±8.85 | 6.46±0.48 |
|  |  | 6 days | 149.16±8.27 | 745.68±29.53 | 252.83±25.20 | 6.63±0.10 |
|  |  | 8 days | 156.28±8.09 | 641.97±45.34 | 273.71±3.44 | 4.96±0.07 |
| NaSA | 50 μM | 24 h | 191.57±17.25 | 685.75±16.66 | 248.80±13.47 | 8.57±0.42 |
|  |  | 48 h | 257.09±1.17 | 891.53±19.59 | 285.87±21.06 | 9.23±0.34 |
|  |  | 4 days | 320.60±28.51 | 831.77±43.70 | 260.61±2.22 | 7.92±1.88 |
|  |  | 6 days | 256.70±19.75 | 905.88±10.34 | 284.37±0.87 | 7.58±0.32 |
|  |  | 8 days | 273.97±19.46 | 1129.14±13.67 | 293.21±13.55 | 5.73±0.19 |
|  | 100 μM | 24 h | 285.64±10.77 | 1131.33±9.85 | 227.66±2.50 | 5.25±0.15 |
|  |  | 48 h | 302.03±6.26 | 797.88±15.80 | 238.57±32.06 | 6.09±0.03 |
|  |  | 4 days | 185.31±14.68 | 718.18±20.09 | 268.30±7.50 | 6.22±0.12 |
|  |  | 6 days | 197.43±13.52 | 861.78±25.76 | 240.60±3.66 | 5.20±0.06 |
|  |  | 8 days | 167.32±9.32 | 917.40±21.84 | 233.17±15.76 | 3.57±0.21 |
| YeE | 1 mg/mL | 24 h | 136.20±8.19 | 697.55±15.54 | 204.71±9.04 | 6.22±0.20 |
|  |  | 48 h | 160.50±21.68 | 386.10±27.26 | 202.53±1.76 | 4.74±0.27 |
|  |  | 4 days | 184.09±0.31 | 574.02±3.14 | 265.89±7.61 | 3.84±0.23 |
|  |  | 6 days | 177.66±18.08 | 472.18±19.39 | 233.84±18.56 | 1.97±0.12 |
|  |  | 8 days | 94.99±6.01 | 824.18±25.57 | 202.32±20.25 | 2.78±0.03 |
|  | 3 mg/mL | 24 h | 116.28±9.80 | 633.58±0.16 | 236.59±3.49 | 6.53±0.28 |
|  |  | 48 h | 155.27±11.81 | 378.31±29.77 | 225.61±10.93 | 4.34±0.39 |
|  |  | 4 days | 182.51±15.38 | 516.36±31.11 | 255.84±15.47 | 2.39±0.10 |
|  |  | 6 days | 274.11±10.72 | 511.27±36.27 | 210.84±18.09 | 1.98±0.02 |
|  |  | 8 days | 115.35±35.98 | 565.48±19.11 | 131.89±5.16 | 2.22±0.04 |

**Table S3.** The amounts of main phenolic compounds (mg/100 g DW±SD) of extracts from *N. officinale* control and experimental in vitro cultures after elicitation (*p* < 0.05 vs control, n=6).

| **Elicitor** | **Elicitor concentration** | **Harvest time after elicitor treatments** | ***p*-Coumaric acid** | **Ferulic acid** | **Rutoside** |
| --- | --- | --- | --- | --- | --- |
| C | 0 | 24 h | 59.00±0.69 | 11.49±1.47 | 7.96±0.34 |
|  |  | 48 h | 4.36±0.66 | 1.39±0.01 | 1.26±0.04 |
|  |  | 4 days | 6.52±0.06 | 5.74±0.10 | 9.76±3.22 |
|  |  | 6 days | 18.53±2.38 | 12.54±1.67 | 21.17±2.67 |
|  |  | 8 days | 64.38±9.26 | 16.46±0.09 | 9.71±3.24 |
| ETH | 25 μM | 24 h | 6.94±0.36 | 11.50±1.17 | 6.57±0.04 |
|  |  | 48 h | 20.09±4.00 | 4.68±1.91 | 1.14±0.23 |
|  |  | 4 days | 11.90±0.59 | 6.43±0.05 | 7.91±0.30 |
|  |  | 6 days | 23.60±4.56 | 9.59±1.67 | 9.58±0.67 |
|  |  | 8 days | 18.96±2.77 | 8.00±0.01 | 15.31±0.78 |
|  | 50 μM | 24 h | 1.80±0.39 | 8.76±0.03 | 2.35±1.54 |
|  |  | 48 h | 16.23±0.86 | 1.72±0.01 | 2.21±1.06 |
|  |  | 4 days | 4.54±0.15 | 9.18±0.14 | 6.66±0.07 |
|  |  | 6 days | 11.05±2.27 | 9.19±1.45 | 12.80±0.26 |
|  |  | 8 days | 15.70±4.07 | 6.52±2.63 | 10.00±0.41 |
| MeJA | 50 μM | 24 h | 15.81±2.16 | 6.09±0.19 | 3.65±0.33 |
|  |  | 48 h | 21.72±0.14 | 5.13±1.34 | 4.18±0.32 |
|  |  | 4 days | 13.47±0.12 | 4.22±0.03 | 3.87±0.01 |
|  |  | 6 days | 3.84±0.50 | 5.62±0.37 | 3.51±0.13 |
|  |  | 8 days | 8.63±0.73 | 7.00±1.15 | 7.73±0.27 |
|  | 100 μM | 24 h | 12.72±0.14 | 6.41±0.45 | 7.72±0.12 |
|  |  | 48 h | 18.71±0.16 | 6.68±0.36 | 6.11±0.01 |
|  |  | 4 days | 5.26±0.15 | 2.75±0.05 | 1.79±0.10 |
|  |  | 6 days | 38.76±0.72 | 7.88±1.11 | 9.37±0.15 |
|  |  | 8 days | 16.09±0.10 | 8.90±0.04 | 7.39±0.11 |
| NaSA | 50 μM | 24 h | 22.93±0.43 | 12.82±0.62 | 16.60±0.08 |
|  |  | 48 h | 4.09±0.29 | 5.22±0.13 | 5.82±1.27 |
|  |  | 4 days | 7.56±0.41 | 8.43±0.32 | 3.21±0.02 |
|  |  | 6 days | 10.24±1.37 | 8.88±0.57 | 11.41±1.07 |
|  |  | 8 days | 14.12±2.25 | 17.76±2.07 | 9.24±1.36 |
|  | 100 μM | 24 h | 22.56±1.84 | 16.30±3.90 | 8.65±3.13 |
|  |  | 48 h | 12.72±0.20 | 6.49±0.19 | 4.50±1.71 |
|  |  | 4 days | 5.11±0.29 | 5.14±0.17 | 2.92±0.06 |
|  |  | 6 days | 10.07±0.23 | 12.97±0.20 | 2.18±0.11 |
|  |  | 8 days | 11.42±1.48 | 12.15±1.67 | 17.00±1.45 |
| YeE | 1 mg/mL | 24 h | 24.29±0.14 | 2.45±0.15 | 4.11±0.83 |
|  |  | 48 h | 9.80±0.05 | 4.36±0.15 | 1.43±0.03 |
|  |  | 4 days | 6.82±0.03 | 7.55±0.03 | 4.43±0.14 |
|  |  | 6 days | 19.86±1.59 | 14.84±1.18 | 12.27±1.05 |
|  |  | 8 days | 13.35±3.71 | 2.21±0.10 | 6.71±0.03 |
|  | 3 mg/mL | 24 h | 24.50±0.76 | 6.46±0.17 | 2.32±0.25 |
|  |  | 48 h | 11.86±3.34 | 9.97±1.10 | 2.91±0.49 |
|  |  | 4 days | 7.74±0.01 | 6.21±0.03 | 5.65±0.52 |
|  |  | 6 days | 9.95±0.63 | 13.67±0.61 | 11.43±0.45 |
|  |  | 8 days | 11.00±0.95 | 9.63±1.88 | 7.41±3.90 |

**Table S4.** Content of photosynthetic pigments (mg/100 g DW±SD) of extracts from *N. officinale* control and experimental in vitro cultures after elicitation (*p* < 0.05 vs control, n=6).

| **Elicitor** | **Elicitor concentration** | **Harvest time after elicitor treatments** | **Chlorophyll *a*** | **Chlorophyll *b*** | **Chlorophyll *a*+*b*** | **Carotenoids** |
| --- | --- | --- | --- | --- | --- | --- |
| C | 0 | 24 h | 12.33±0.33 | 13.31±0.41 | 25.64±0.73 | 2.42±0.01 |
|  |  | 48 h | 4.38±0.65 | 1.56±0.08 | 5.99±0.81 | 0.69±0.01 |
|  |  | 4 days | 85.56±10.51 | 56.00±5.54 | 141.50±15.97 | 9.33±1.79 |
|  |  | 6 days | 48.11±13.12 | 72.94±40.90 | 121.16±54.02 | 0.00±0.00 |
|  |  | 8 days | 13.37±5.21 | 16.88±7.74 | 30.25±12.96 | 1.67±0.06 |
| ETH | 25 μM | 24 h | 24.37±0.24 | 22.87±0.73 | 47.24±0.98 | 3.46±0.16 |
|  |  | 48 h | 36.07±0.98 | 27.94±1.39 | 64.01±2.20 | 4.03±0.16 |
|  |  | 4 days | 47.53±13.44 | 36.18±10.92 | 83.71±24.36 | 4.49±1.14 |
|  |  | 6 days | 26.16±8.80 | 21.32±8.47 | 47.47±17.27 | 2.65±0.65 |
|  |  | 8 days | 33.36±0.24 | 19.19±0.08 | 52.49±0.24 | 4.44±0.08 |
|  | 50 μM | 24 h | 22.76±3.83 | 15.21±2.93 | 37.97±6.76 | 2.54±0.33 |
|  |  | 48 h | 62.80±8.47 | 49.61±6.60 | 112.41±15.07 | 7.37±1.14 |
|  |  | 4 days | 74.09±9.94 | 48.57±5.46 | 122.60±15.32 | 8.24±1.22 |
|  |  | 6 days | 23.28±6.52 | 18.78±5.05 | 42.06±11.57 | 3.23±1.14 |
|  |  | 8 days | 40.39±3.18 | 21.84±0.90 | 62.17±4.16 | 5.99±0.98 |
| MeJA | 50 μM | 24 h | 45.57±6.76 | 35.09±7.09 | 80.72±13.93 | 4.90±0.41 |
|  |  | 48 h | 47.59±7.82 | 34.34±7.50 | 81.99±15.24 | 5.47±0.41 |
|  |  | 4 days | 39.58±4.32 | 28.12±3.91 | 67.70±8.23 | 5.82±0.57 |
|  |  | 6 days | 14.23±1.87 | 10.60±2.44 | 24.89±4.24 | 2.88±0.16 |
|  |  | 8 days | 18.32±2.61 | 11.12±1.55 | 29.38±4.24 | 5.70±0.41 |
|  | 100 μM | 24 h | 76.05±15.81 | 51.57±9.21 | 127.56±24.93 | 8.01±2.04 |
|  |  | 48 h | 73.29±2.61 | 49.55±3.91 | 122.89±6.60 | 8.30±0.33 |
|  |  | 4 days | 34.63±0.90 | 27.08±0.81 | 61.71±1.71 | 4.95±0.33 |
|  |  | 6 days | 14.75±2.44 | 11.87±2.44 | 26.62±4.89 | 2.42±0.01 |
|  |  | 8 days | 21.49±0.08 | 14.35±1.87 | 35.84±1.96 | 5.07±0.33 |
| NaSA | 50 μM | 24 h | 52.03±3.99 | 35.78±3.67 | 87.80±7.66 | 5.70±0.41 |
|  |  | 48 h | 55.77±1.79 | 37.74±2.53 | 93.57±4.40 | 7.32±0.08 |
|  |  | 4 days | 54.56±0.41 | 33.99±0.33 | 88.55±0.73 | 6.86±0.08 |
|  |  | 6 days | 60.38±3.26 | 38.03±1.14 | 98.35±4.48 | 8.30±0.65 |
|  |  | 8 days | 73.80±4.64 | 39.47±1.71 | 113.27±6.36 | 10.83±1.14 |
|  | 100 μM | 24 h | 89.01±2.53 | 58.25±1.87 | 147.32±4.48 | 8.47±0.24 |
|  |  | 48 h | 69.48±1.63 | 45.29±1.14 | 114.83±0.41 | 6.91±1.14 |
|  |  | 4 days | 51.33±1.39 | 32.78±0.73 | 84.12±0.65 | 5.53±1.47 |
|  |  | 6 days | 76.28±4.24 | 49.66±6.36 | 126.06±10.59 | 8.70±0.90 |
|  |  | 8 days | 76.86±4.24 | 44.25±1.47 | 121.11±5.70 | 10.02±1.14 |
| YeE | 1 mg/mL | 24 h | 28.00±2.28 | 28.46±3.42 | 56.40±5.62 | 3.05±0.08 |
|  |  | 48 h | 11.70±0.08 | 9.79±0.33 | 21.55±0.16 | 1.84±0.16 |
|  |  | 4 days | 33.94±3.99 | 25.58±3.91 | 59.46±7.82 | 3.69±0.16 |
|  |  | 6 days | 9.39±0.24 | 8.70±0.08 | 18.09±0.33 | 2.25±0.08 |
|  |  | 8 days | 45.80±4.64 | 30.13±4.81 | 75.94±9.45 | 6.16±0.57 |
|  | 3 mg/mL | 24 h | 26.21±0.57 | 27.02±0.57 | 53.18±1.22 | 2.54±0.01 |
|  |  | 48 h | 12.56±1.47 | 11.52±0.65 | 24.08±2.12 | 1.73±0.16 |
|  |  | 4 days | 15.21±1.14 | 12.62±0.57 | 27.89±1.79 | 3.80±0.33 |
|  |  | 6 days | 5.59±1.06 | 7.03±5.21 | 12.62±6.27 | 1.04±0.81 |
|  |  | 8 days | 17.05±0.49 | 15.67±0.98 | 32.73±1.47 | 2.94±0.08 |

**Table S5.** Antioxidant activity estimated by CUPRAC, DPPH and FRAP methods (expressed in mmol TE/100 g DW±SD) of extracts from *N. officinale* control and experimental in vitro cultures after elicitation (*p* < 0.05 vs control, n=6).

| **Elicitor** | **Elicitor concentration** | **Harvest time after elicitor treatments** | **CUPRAC** | **DPPH** | **FRAP** |
| --- | --- | --- | --- | --- | --- |
| C | 0 | 24 h | 1.63±0.06 | 1.19±0.03 | 0.40±0.01 |
|  |  | 48 h | 0.84±0.13 | 0.94±0.05 | 0.16±0.01 |
|  |  | 4 days | 2.61±0.02 | 0.94±0.07 | 0.75±0.01 |
|  |  | 6 days | 2.27±0.03 | 1.04±0.01 | 0.72±0.01 |
|  |  | 8 days | 1.31±0.03 | 0.74±0.03 | 0.44±0.01 |
| ETH | 25 μM | 24 h | 1.67±0.03 | 0.68±0.01 | 0.27±0.01 |
|  |  | 48 h | 1.46±0.02 | 0.63±0.05 | 0.27±0.01 |
|  |  | 4 days | 2.18±001 | 0.85±0.09 | 0.63±0.01 |
|  |  | 6 days | 1.79±0.11 | 0.79±0.12 | 0.54±0.01 |
|  |  | 8 days | 1.64±0.21 | 0.73±0.11 | 0.37±0.06 |
|  | 50 μM | 24 h | 1.77±0.65 | 0.53±0.05 | 0.16±0.01 |
|  |  | 48 h | 2.12±0.12 | 0.54±0.01 | 0.36±0.02 |
|  |  | 4 days | 2.69±0.07 | 0.81±0.03 | 0.70±0.01 |
|  |  | 6 days | 1.94±0.10 | 2.30±2.09 | 0.59±0.02 |
|  |  | 8 days | 1.69±0.07 | 0.61±0.01 | 0.40±0.08 |
| MeJA | 50 μM | 24 h | 2.42±0.08 | 0.73±0.03 | 0.76±0.05 |
|  |  | 48 h | 2.03±0.07 | 0.70±0.05 | 0.52±0.01 |
|  |  | 4 days | 2.07±0.03 | 0.69±0.01 | 0.54±0.01 |
|  |  | 6 days | 1.41±0.01 | 0.58±0.10 | 0.32±0.01 |
|  |  | 8 days | 1.56±0.26 | 0.68±0.11 | 0.36±0.07 |
|  | 100 μM | 24 h | 2.97±0.03 | 0.96±0.04 | 0.83±0.02 |
|  |  | 48 h | 2.54±0.05 | 0.69±0.09 | 0.66±0.01 |
|  |  | 4 days | 1.62±0.02 | 0.68±0.11 | 0.35±0.01 |
|  |  | 6 days | 1.40±0.02 | 0.65±0.01 | 0.31±0.01 |
|  |  | 8 days | 1.73±0.02 | 0.59±0.06 | 0.41±0.01 |
| NaSA | 50 μM | 24 h | 2.16±0.03 | 0.67±0.04 | 0.61±0.01 |
|  |  | 48 h | 2.08±0.06 | 0.94±0.01 | 0.64±0.01 |
|  |  | 4 days | 2.48±0.44 | 0.86±0.07 | 0.60±0.03 |
|  |  | 6 days | 2.24±0.06 | 0.88±0.02 | 0.62±0.01 |
|  |  | 8 days | 2.52±0.01 | 0.94±0.10 | 0.72±0.02 |
|  | 100 μM | 24 h | 2.64±0.04 | 0.94±0.10 | 0.79±0.04 |
|  |  | 48 h | 2.13±0.01 | 0.91±0.02 | 0.59±0.02 |
|  |  | 4 days | 2.02±0.05 | 0.99±0.19 | 0.50±0.01 |
|  |  | 6 days | 2.30±0.01 | 0.83±0.02 | 0.54±0.01 |
|  |  | 8 days | 2.32±0.07 | 0.92±0.12 | 0.53±0.01 |
| YeE | 1 mg/mL | 24 h | 1.88±0.67 | 0.81±0.26 | 0.17±0.01 |
|  |  | 48 h | 1.26±0.04 | 0.94±0.24 | 0.23±0.03 |
|  |  | 4 days | 1.99±0.03 | 0.87±0.01 | 0.51±0.01 |
|  |  | 6 days | 1.41±0.07 | 0.92±0.01 | 0.42±0.02 |
|  |  | 8 days | 1.76±0.01 | 1.00±0.17 | 0.31±0.01 |
|  | 3 mg/mL | 24 h | 1.51±0.01 | 0.62±0.09 | 0.19±0.01 |
|  |  | 48 h | 1.34±0.01 | 0.83±0.03 | 0.23±0.03 |
|  |  | 4 days | 1.64±0.09 | 1.05±0.11 | 0.48±0.02 |
|  |  | 6 days | 1.48±0.35 | 1.17±0.05 | 0.36±0.01 |
|  |  | 8 days | 1.02±0.01 | 1.00±0.47 | 0.15±0.01 |
